# Hemispheric Cortical, Cerebellar and Caudate Atrophy Associated to Cognitive Impairment in Metropolitan Mexico City Young Adults Exposed to Fine Particulate Matter Air Pollution

**DOI:** 10.3390/toxics10040156

**Published:** 2022-03-25

**Authors:** Lilian Calderón-Garcidueñas, Jacqueline Hernández-Luna, Partha S. Mukherjee, Martin Styner, Diana A. Chávez-Franco, Samuel C. Luévano-Castro, Celia Nohemí Crespo-Cortés, Elijah W. Stommel, Ricardo Torres-Jardón

**Affiliations:** 1 College of Health, The University of Montana, Missoula, MT 59812, USA; 2 Escuela de Ciencias de la Salud, Universidad del Valle de México, Mexico City 14370, Mexico; alejeje01@gmail.com (D.A.C.-F.); y2j.luevano@hotmail.com (S.C.L.-C.); celia.crespo@uvmnet.edu (C.N.C.-C.); 3 Radiology Department, Hospital HMG, Coyoacán, Mexico City 04380, Mexico; radiologa.jacqueline.hernandez@gmail.com; 4 Interdisciplinary Statistical Research Unit, Indian Statistical Institute, Kolkata 700108, India; psmukherjee.statistics@gmail.com; 5 Neuro Image Research and Analysis Lab, University of North Carolina, Chapel Hill, NC 27599, USA; martin.styner@mac.com; 6 Department of Neurology, Geisel School of Medicine at Dartmouth, Hanover, NH 03755, USA; elijah.w.stommel@hitchcock.org; 7 Instituto de Ciencias de la Atmósfera y Cambio Climático, Universidad Nacional Autónoma de México, Mexico City 04510, Mexico; rtorres@unam.mx

**Keywords:** Alzheimer, air pollution, brain atrophy, caudate and cerebellar atrophy, cognition, MOCA, MRI, Mexico City, Parkinson, PM_2.5_

## Abstract

Exposures to fine particulate matter PM_2.5_ are associated with Alzheimer’s, Parkinson’s (AD, PD) and TDP-43 pathology in young Metropolitan Mexico City (MMC) residents. High-resolution structural T1-weighted brain MRI and/or Montreal Cognitive Assessment (MoCA) data were examined in 302 volunteers age 32.7 ± 6.0 years old. We used multivariate linear regressions to examine cortical surface area and thickness, subcortical and cerebellar volumes and MoCA in ≤30 vs. ≥31 years old. MMC residents were exposed to PM_2.5_ ~ 30.9 µg/m^3^. Robust hemispheric differences in frontal and temporal lobes, caudate and cerebellar gray and white matter and strong associations between MoCA total and index scores and caudate bilateral volumes, frontotemporal and cerebellar volumetric changes were documented. MoCA LIS scores are affected early and low pollution controls ≥ 31 years old have higher MoCA vs. MMC counterparts (*p* ≤ 0.0001). Residency in MMC is associated with cognitive impairment and overlapping targeted patterns of brain atrophy described for AD, PD and Fronto-Temporal Dementia (FTD). MMC children and young adult longitudinal studies are urgently needed to define brain development impact, cognitive impairment and brain atrophy related to air pollution. Identification of early AD, PD and FTD biomarkers and reductions on PM2.5 emissions, including poorly regulated heavy-duty diesel vehicles, should be prioritized to protect 21.8 million highly exposed MMC urbanites.

## 1. Introduction

In the scenario of severe exposures to air pollutants, starting in utero [1], metropolitan Mexico City (MMC) young residents exhibit infra and supratentorial aberrant hyperphosphorylated tau (p-τ), β- amyloid, α synuclein and TAR DNA-binding protein-43 (TDP-43) pathology [2,3]. These quadruple aberrant proteins are identified starting in pediatric ages and are associated with the presence of highly oxidative nanoparticles (NPs) resulting from fossil fuels’ combustion, engineered nanoparticles, forest wildfires, volcanic eruptions, etc., [3,4]. Evidence from epidemiological studies points to fine particulate matter (PM_2.5_) increasing the risk of dementia [5,6,7,8,9,10], and negatively impacting cognitive abilities in children and adolescents [11,12].

We have reported fluid and crystallized cognition deficits in MMC children cohorts age 6.9 ± 0.67 years old and 10.7 ± 2.7 years old, versus low-pollution matched children, along MRI prefrontal white matter hyperintense lesions, white matter volume differences in bitemporal and right parietal and no changes in the subcortical regions, including the hippocampus, caudate, putamen, globus pallidus and amygdala [13,14].We have also examined 134 consecutive MMC forensic autopsies in subjects ≤ 30 years, average age 20.03 ± 6.38 years (range 11 months to 30 years) and documented neuropathological hallmarks of Alzheimer’s disease (AD) in 99% of autopsies, and in a simultaneous clinical study of 150 seemingly healthy young urbanites age 21.6 ± 3.5 y, we reported cognitive impairment in 66% of them using the Montreal Cognitive Assessment instrument (MoCA) [15]. In a MoCA study of 517 urbanites, average age 21.6 ± 5.8 years old exposed to PM_2.5_ in various Mexican polluted cities, 55% of the population was cognitively impaired with scores ≤ 25 (normal 26–30) [16]. In sharp contrast, MMC residents ≥ 31 years old had MoCA scores on average 20.4 ± 3.4 vs. low pollution controls 25.2 ± 2.4 (*p* < 0.0001) [17]. The information from these Mexican cognitive studies suggested that brain volumetric changes were significant beyond age 31, and since COVID restricted our capacity to have brain MRI volunteers in a low pollution city, we decided to compare ≥31 years vs. ≤30 years old subjects while we wait for the opportunity to screen the controls.

Brain structural MRI allows for the noninvasive assessment of cortical and subcortical morphology. Hemispheric and subcortical regional specificity of targeted brain structures affected by air pollutant exposure is at the core of our interest in MMC highly exposed populations, knowing that in forensic samples with no extra-neural pathology, 99.5% of individuals 40 and younger have Alzheimer’s disease (AD) hallmarks, 23% have Parkinson’s disease (PD) and 18% TDP-43 pathology. In this study, we focused on MMC healthy middle-class college educated volunteers and used brain cortical thickness, surface area, subcortical volumes and MoCA results to compare individuals ≤30 versus ≥31 years old with a lifetime residency in one of the most polluted cities in North America. We hypothesized that lifetime PM_2.5_ exposures would be associated with progressive regional brain changes and worse cognitive performance. We report significant differences in gray, white matter and CSF volumes, regional cortical thickness, cortical surface area, caudate and cerebellar volumes between MMC residents younger than 30 years versus older than 31 years and provide cognitive correlates taking into account formal education years, sex and age.

## 2. Materials and Methods

### 2.1. Study Population, Inclusion and Exclusion Criteria

This 2018–2020 protocol was conducted according to the Revised Helsinki Declaration of 2000 and approved by the review boards and ethics committees at the Universidad del Valle de Mexico (16 March 2016) and the University of Montana (IRB# 206 R-09 and 185-20). Written consent was obtained from the adult volunteers and from parents of minors, who also signed a verbal consent. This study was aimed to middle class volunteers and was advertised by word of mouth among families involved in previous clinical studies and through social networking. The participating 304 right-handed volunteers included: (1) thirty-four MMC volunteers with brain MRI/MoCA, (2) MMC only MoCA 233 volunteers, and (3) control, low pollution city (Hermosillo) MoCA 35 volunteers. Subjects completed a baseline examination and were considered clinically healthy. Briefly, inclusion criteria involved each volunteer being able to validly and safely complete the clinical visits and MoCA and/or brain MRI studies, negative smoking history and environmental tobacco/nicotine exposure, lifelong residency in MMC or the control city, residency within a diameter of 5 km of the closest monitoring station from their residence, and unremarkable clinical histories, including the absence of a history of hospitalizations for respiratory illnesses, ear-nose-throat (ENT) and oral symptomatology and/or surgery, head trauma, systemic or respiratory viral diseases, cardiovascular diseases, diabetes, metabolic syndrome, obesity, and family histories of Alzheimer’s, Parkinson’s, frontotemporal dementia (FTD) and amyotrophic lateral sclerosis (ALS). We specifically excluded subjects with active participation in team sports, head trauma, who had occupational exposures to toxic substances and who had a history of drug abuse, including alcohol and marijuana. Sporadic, social intake of alcohol was recorded in 12% of participant adults. All included subjects were taking no medications, slept in bedrooms with no carpeting and had open windows for ventilation, had kitchens separated from living and sleeping areas and used gas for cooking. We examined cross-sectional regional and hemispheric specific differences in brain morphometry and cognition in Metropolitan Mexico City ≤30 years old (average 22 ± 3.3 years) (n: 20) versus MMC volunteers ≥ 31 years old (average age 42.7 ± 9.3 years) (n: 14) and included MoCA only data from MMC and Hermosillo controls for MoCA scores comparison.

### 2.2. Brain MRI Acquisition and Processing

The 3D MRI for all subjects was acquired on a 1.5 Tesla 5T Signa Excite HD MR (General Electric Milwaukee WI, USA) with an 8 Channel Brain Array. High-resolution T1 weighted anatomical images were acquired, as well as T2 weighted images using a 2D multi-slice dual fast spin echo sequence and fluid attenuated inversion recovery images. All sequences covered the entire surface of the brain. Atlas moderated probabilistic tissue segmentations of white matter, gray matter and cerebrospinal fluid were computed in automatic fashion including skull stripping, intensity inhomogeneity correction and intensity calibration. Next, subcortical structures and 90 cortical parcellations (Neuromorphometrics Inc., Somerville, MA, USA) were determined via a multi-atlas multi-modality segmentation process. The parcellation for each subject was then combined with that subject’s tissue classification to obtain white matter, gray matter and cerebral-spinal fluid volumes for each region. The corresponding volumetric measurements for ROIs and parcellations were automatically computed. Cortical surface reconstruction and subcortical segmentation were accomplished via FreeSurfer (version 5.3) including total gray and white matter and subcortical volumes, cortical thickness and cortical surface area estimates for cortical regions via the Desikan-Killiany Atlas [18,19]. At the Neuro Image Research and Analysis Lab, University of Carolina at Chapel Hill, T1-weighted structural images underwent quality control (QC) across five categories, both prior to and after post-processing to gauge intensity inhomogeneity, white matter underestimation, pial overestimation, and magnetic susceptibility artefact [19]. Only images passing QC for all categories were included in this study. In summary, measurements on structural MRI data included (i) whole brain tissue volumes of white matter and gray matter, CSF, (ii) cortical regions and quantification of WM/GM/CSF, (iii) measurements of all major sub-cortical structures, including the hippocampus, amygdala, caudate, putamen, globus pallidus and lateral ventricles, and (iv) cortical thickness for whole brain and lobar parcellations.

### 2.3. Calculation of Accumulated PM_2.5_ Exposure

We obtained a detailed residency and exposure history from each volunteer. The collected data consisted of historical 24-h average PM_2.5_ mass concentrations sampled at the closest monitoring station to their residence. The PM_2.5_ data were collected from the air quality-monitoring network of Mexico City’s Secretariat of the Environment under a six-day schedule following the recommendations of the US EPA for PM samplers [20]. Given that systematic PM_2.5_ measurements were not available before 2004, fine particle concentrations PM_2.5_ for the period 1989 to 2004 were estimated from PM_10_ measurements performed in the same monitoring sites for that period. The associated PM_2.5_ levels were then estimated from the average of the slopes of the linear regression analysis of the 24-h means of PM_10_ and PM_2.5_ for each year and site from 2004 to 2008. Neither PM_10_ nor PM_2.5_ data are available before 1989. Thus, we assumed that the estimated PM_2.5_ level for previous years was equivalent to the value reported for the year 1989. Upon entrance to the study, the geolocation of the subject residency, school and workplace were mapped out and the cumulative exposure of the PM_2.5_ was calculated and integrated into the database. The cumulative exposure was obtained by summing up the annual average PM_2.5_ mass concentration in excess to the US EPA annual NAAQS from the year of the test (2019) and backwards up to 1989. We applied the current primary US EPA annual standard for PM_2.5_ stands for an annual mean of 12.0 µg/m^3^ averaged over three years.

### 2.4. Neurocognitive Performance

The Spanish version of MoCA was used in this study (Certification MXCRECE191274-01). The MMC 34 brain MRI volunteers plus 233 MMC subjects and 35 low pollution controls underwent MoCA testing. MoCA assesses global cognitive function and contains 10 subtests [21,22,23,24,25,26]. MoCA scores were converted into six index scores based on the combinations used by Julayanont et al. and Petersen et al. [24,26].

### 2.5. Covariates

The following covariates were included in the main analysis: age, sex, years of formal education, BMI, MoCA scores, cognition indexes, and socioeconomic status (SES). For subcortical volume analysis, intracranial volume (ICV) was a covariate.

### 2.6. Study City and Air Quality

The MMC area covers ~7585 km^2^ and is located on an elevated basin 2240 m above sea level surrounded by mountain ridges on three sides. MMC has a population of ~21.8 million people. Emissions from ~five million vehicles, over 50,000 industries and LP gas, industrial and household solvents, and vapors of oil derived liquid fuels combine with high solar radiation and poor ventilation to produce a severe air pollution problem with a strong oxidizing capacity [27,28,29,30]. MMC residents have been exposed to high levels of primary fine and ultrafine particles as well as secondary air pollutants including secondary organic aerosols and ozone concentrations at levels above United States National Air Ambient Quality Standards (NAAQS) all year round during the last two decades [20,27,28,29,30]. High levels of black carbon (BC), polycyclic aromatic hydrocarbons (PAHs), semi-volatile organic compounds from incomplete combustion of carbonaceous fuels such as gasoline and diesel, as well as metals from brake and tire wear have been historically found in the PM_2.5_ fraction of MMC [31,32,33,34,35]. As a result, MMC residents, including children and pregnant women, are exposed to outdoor elevated NP concentrations rich in PAHs and metals [36,37]. Commuting in any of the urban transport modes available in the urban area is also associated with high NPs exposures [38]. Traveling in the MMC subway system results in high PM_2.5_ exposures between 34 and 93 μg m^−3^; equivalent PAHs concentrations ranging from 19 to 41 ng m^−3^; and NPs up to 50,300 ± 10,600 (# cm^−3^) with an average size of 38.5 ± 15.9 nm, and elevated concentrations of Fe, Cu, Ni, Cr and Mn [38,39]. Metals present in the underground subway are the result of friction, brake wear, and sparking from rail grinding. Ozone has been also above the US EPA standards, with higher concentrations in Southwest MMC [28,33].

Hermosillo, the selected control city, is located in the southern extreme of the Sonora Desert in northwestern Mexico. It has a population of nearly 813,000 people and an urban area of around 168 km^2^ (centroid radius of ~7.4 km). The climate is dry and characterized by arid to semiarid conditions with good wind ventilation conditions. The urban area has diverse emission sources of air pollutants, including traffic, domestic combustion and industry, and is surrounded by areas with strong agricultural activities. Most streets are unpaved and, as a result, the re-suspension of dusts with dominant particulate matter ≥2.5 μm constitutes the main air pollution problem [40,41]. Several heavy metals have been identified in dust, and lead seems to be the most important [42]. The anthropogenic lead content in the dust is greater than the geogenic one, suggesting that since leaded-gasoline has not been used in Mexico in the last 30 years, this signature shows a Pb-legacy. Lead chromate (crocoite) from yellow paint in the inhalable fraction of dusts has also been reported [43]. However, the Pb PM concentration does not exceed the respective NAAQS standard (0.15 μg/m^3^ of lead in total suspended particles (TSP) in a 3-month average) [42]. The control city has concentrations of PM_2.5_, SO_2_, NO_2_, CO, SO_2_ and O_3_ below the respective US EPA NAAQS short- and long-term exposures [44].

### 2.7. Statistical Analysis

We first calculated the sample mean and sample standard deviation of the total MoCA score in each group including Non-MRI-MMC age ≤ 30 years, Non-MRI-MMC age ≥ 31 years, MRI-MMC age ≤ 30 years, MRI-MMC age ≥ 31 years, Hermosillo age ≤ 30 years, and Hermosillo age ≥ 31 years and included Cognitive Domain scores and index scores (Supplemental Tables S1 and S2). Next, we performed multiple linear regressions to test the significance of mean difference of various group scores after adjusting age, gender, BMI and education years. We consider these pairs of groups: (i) Non-MRI-MMC age ≤ 30 years and Hermosillo age ≤ 30 years, (ii) Non-MRI-MMC age ≥ 31 years and Hermosillo age ≥ 31 years, (iii) MRI-MMC age ≤ 30 years and Hermosillo age ≤ 30 years, and (iv) MRI-MMC age ≥ 31 years and Hermosillo age ≥ 31 years. The adjusted *p*-values are reported in Supplemental Tables S1 and S2. Between-group differences in 34 right- handed subjects (≤30 y vs. ≥31 y) were assessed using multivariable linear regressions of the pooled means of regional cortical thickness (mm), regional and total cortical surface area (mm^2^), regional subcortical volume on the age-group, and intracranial volume (ICV; mm^3^) as predictors. P values were corrected for false discovery rate (FDR). We also performed multivariate linear regression of MoCA index scores on regional cerebral volumes and cortical thickness, age and intracranial volume, and calculated *p*-values for the significance of the linear association between various MoCA index scores and regional cerebral volumes and cortical thickness. We used MS-Excel and statistical software “R” to perform these analyses.

## 3. Results

### 3.1. Air Pollution

**Figure 1:**
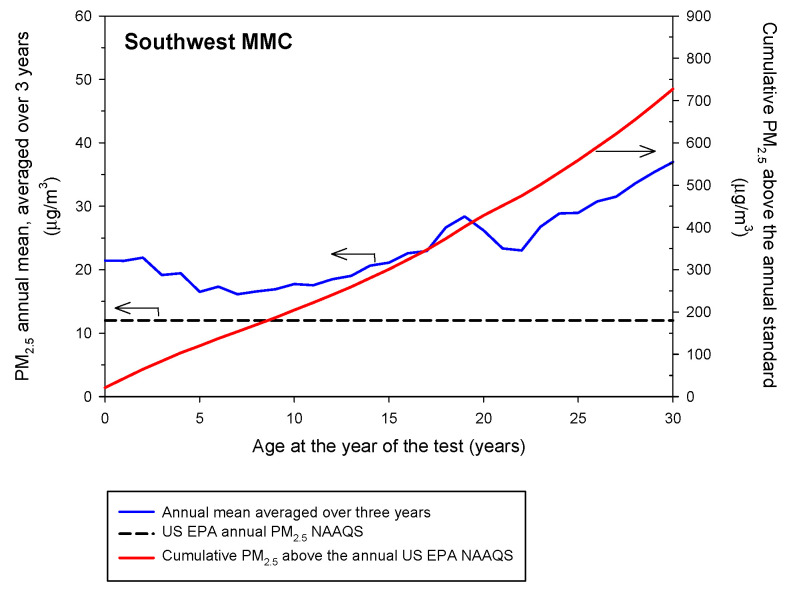
Backward annual trends of the PM_2.5_ annual mean concentrations averaged over three years and the respective cumulative PM_2.5_ concentrations determined for the southwest sector of MMC according to the age of the volunteers starting in 2019. The average annual PM_2.5_ exposure was calculated as 28.36 μg/m^3^/year.

**Figure 2:**
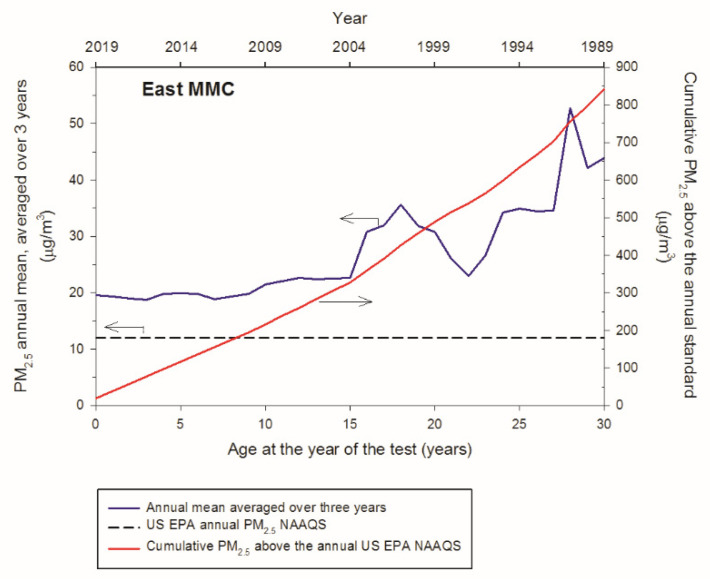
Backward annual trends of the PM_2.5_ annual mean concentrations averaged over three years and the respective cumulative PM_2.5_ concentrations determined for the east sector of MMC according to the age of the volunteers at the year of the study (2019). The average annual PM_2.5_ exposure was calculated as 33.4 μg/m^3^/year.

Twenty-two million MMC residents are exposed to PM_2.5_ above the annual US EPA NAAQS. Figure 1 and Figure 2 show the backward trend of the annual mean concentrations averaged over three years of PM_2.5_ 24-h data and the respective cumulative PM_2.5_ in excess of the annual US EPA NAAQS for Southwest and East MMC sectors in accordance with the volunteers’ age, starting in 2019. Volunteers of age ≤ 30 years old living in either one of the MMC sectors targeted have been exposed to an equivalent average of nearly two times the reference USEPA standard, while volunteers age ≥ 30 years old have been exposed to annual concentrations between 35 and 45 μg/m^3^, three to four times the level allowed in the US. Taking into consideration that the estimated backward trend of cumulative PM_2.5_ mass concentrations for Southwest and East MMC was represented by a straight line, the rates of PM_2.5_ accumulation as people aged would be 28.36 μg/m^3^/year and 33.4 μg/m^3^/year, respectively. Remarkably, North and West MMC sectors register higher cumulative PM_2.5_ levels compared to SW and East MMC, thus displacements of a person within the urban area would not have reduced the level of exposure.

**Figure 3:**
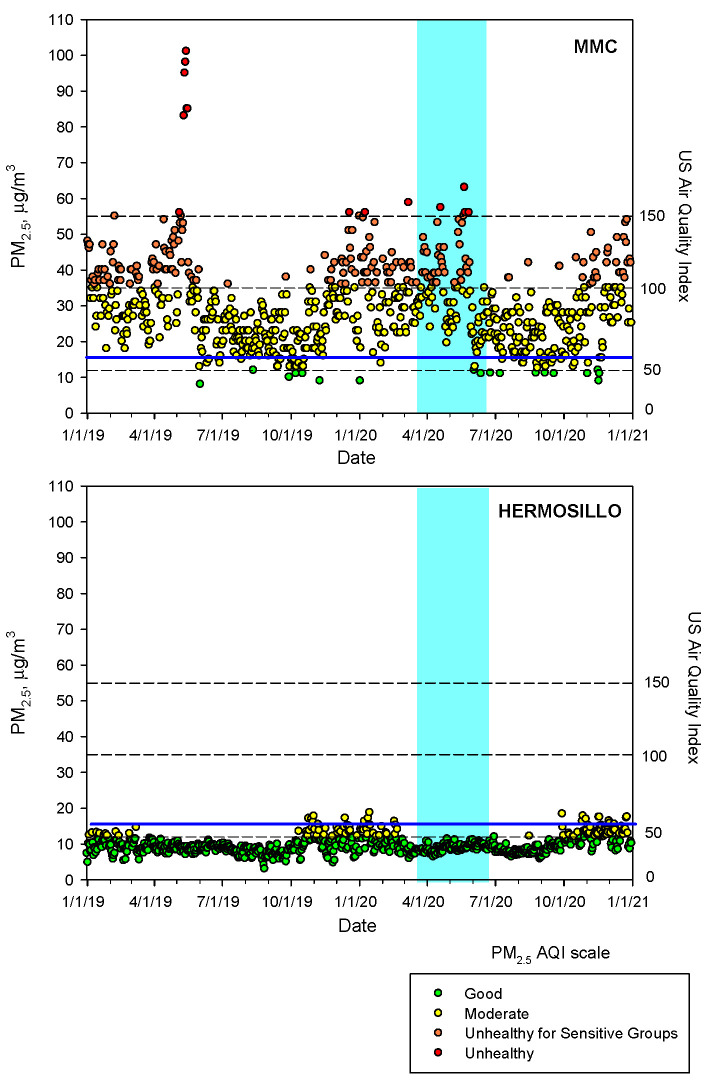
Time-series of maxima PM_2.5_ 24-h averages at MMC and daily means of PM_2.5_ in Hermosillo from 1 January 2019 to 31 December 2020 following the US EPA AQI index. The blue continuous line depicts the revised 24-h average guideline of the WHO. The blue shade area represents the official lockdown COVID-period in Mexico. Air quality data are available from Sistema de Monitoreo Atmosferico del Gobierno de la Ciudad de México (http://www.aire.cdmx.gob.mx/default.php accessed on 18 December 2021) and from Red Universitaria de Observatorios Atmosféricos de la Universidad Nacional Autónoma de México (https://www.ruoa.unam.mx/ accessed on 18 December 2021).

To show the contrast of PM_2.5_ air quality between MMC and Hermosillo, the low pollution control city, we plotted the time series of the maxima PM_2.5_ daily averages registered for the period 2019–2020 (Figure 3). In sharp contrast, Hermosillo has PM_2.5_ concentrations below the USEPA standard versus MMC with unhealthy PM_2.5_ concentrations regardless of pre-COVID-19 and COVID-19 times.

### 3.2. Study Population and Demographics

The participating 302 healthy volunteers included: (1) Thirty-four MRI/MoCA, MMC volunteers, average age 32.4 ± 6.3 years old with 15.2 ± 1.8 years of formal education, and (2) MMC MoCA only 233 volunteers, age: 34.0 ± 7.6 y, and 35 clean air controls, age 31.7 ± 4.2 y (MoCA only 268 subjects) (Table 1).

### 3.3. Total Gray and White Matter Volumes and CSF, Cortical Thickness, Cortical Surface Area, and Intracranial Volume ICV

**Figure 4:**
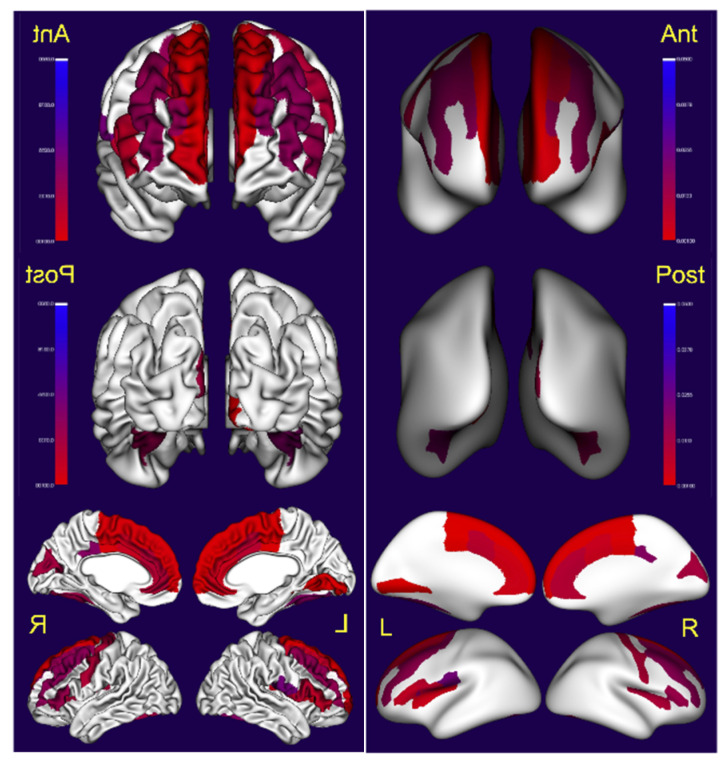
Visualization of the statistical significance of cortical thickness (left) measurements in cortical regions. On the right side, the inflated surface. Non-significant regions are in white, significant regions (following correction for multiple comparison) are shown in blue (*p* < 0.05) to red (*p* < 0.001).

**Figure 5:**
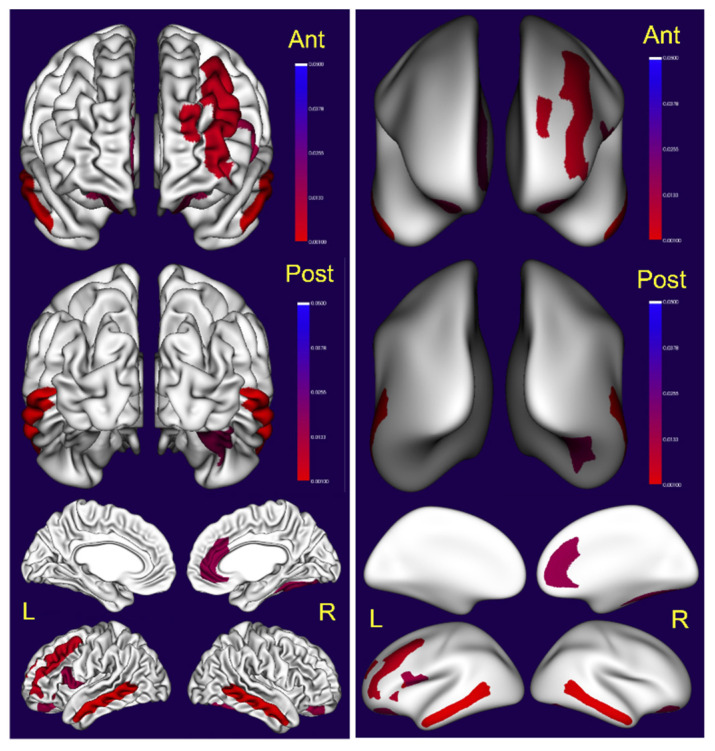
Visualization of the statistical significance of surface area measurements in cortical regions (left). On the right, the inflated surface. Non-significant regions are in white, significant regions (following correction for multiple comparison) are shown in blue (*p* < 0.05) to red (*p* < 0.001).

**Figure 6:**
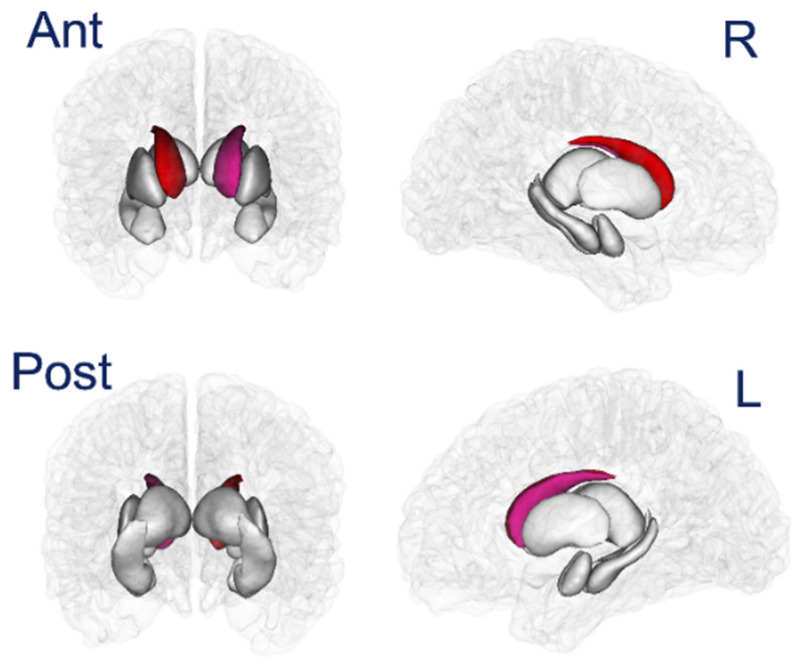
Visualization of the statistical significance of subcortical volumes. Non-significant regions are in white, significant regions (following correction for multiple comparison) are shown in blue (*p* < 0.05) to red (*p* < 0.001).

We found significant differences in total gray matter, CSF volumes, cortical thickness and surface area in supratentorial regions and volume changes in subcortical regions, including cerebellar gray and white matter and bilateral caudate volumes (Table 2, Figure 4, Figure 5 and Figure 6). Indeed, gray matter was significantly decreased in ≥31 years old volunteers *p* < 0.0001 versus ≤30 y, with robust decreases in cortical thickness predominantly in the left hemispheric brain regions, including the temporal middle gyrus, cingulate and frontal superior, middle and inferior lobes (Figure 4, Table 2).

Significant changes in surface areas were also documented (Figure 5).

### 3.4. Subcortical Volume

There was a significant decrease in right cerebellar gray and white matter and left cerebellar gray matter, and a predominant right smaller caudate and lesser left caudate involvement (Figure 6). No differences were documented for the hippocampus, putamen, pallidum, amygdala and nucleus accumbens (Table 2). CSF was significantly increased in ≥31 years old volunteers (*p* = 0.0008).

### 3.5. MoCA Results

MRI/MoCA ≤ 30 years old subjects had on average 24.5 ± 2.6 points, in the range of mild cognitive impairment (MCI), and were not significantly different from MMC only MoCA similar age subjects (*p* = 0.4) or similar age Hermosillo subjects (*p* = 0.4). Strikingly, for the MRI/MoCA ≥ 31 years old volunteers with 16.0 ± 2.1 years of education, their scores were 23.3 ± 2.8 and significantly different from the 20.4 ± 3.4 score in the MoCA only MMC subjects with 13.2 ± 3.3 years of formal education. Outstandingly, low pollution controls had significantly higher MoCA values: 25.2 ± 2.3 versus their MMC ≥ 31 years counterparts (*p* = < 0.0001) (Table 1, Supplemental Tables S1 and S2).

### 3.6. MoCA Total Score, Index Scores, Cortical Thickness and Subcortical Volumes

We analyzed the correlation between MoCA total scores, index scores and the different cortical and subcortical volumes, cortical thickness and surface areas in MMC/MRI ≤ 30 vs. ≥31 years old subjects (Table 3).

Remarkably, MoCA total scores were significantly associated with right and left caudate decreased volume and left orbital gyrus’ surface area. Orientation was associated with the right temporal superior transverse gyrus, in spite orientation scores were marginally significant between groups (Supplemental Table S2). Executive Index Scores (EIS) were strongly associated with decreased volume on the left orbital lateral sulcus, left frontal inferior triangular gyrus and right cerebellar white matter (Table 3). Language Index Scores (LIS) were associated with the right temporal superior transverse gyrus, left gyrus and cingulate middle anterior and posterior sulcus, and left orbital gyrus volume. The Visuospatial index (VIS) was associated with the left orbital lateral sulcus, and the Attention Index Score (AIS) was associated with the right temporal superior transverse gyrus volume. The Summary Score was strongly associated to decreased volume in the orbital left gyrus (*p* = 0.0009).

## 4. Discussion

Lifelong exposures to PM_2.5_ above the current USEPA standard are associated with a significant decrease in gray matter from higher order cortical regions, commonly associated with Alzheimer’s, Parkinson’s and FTD in 42.7 ± 9.3 years old, healthy, college educated, Metropolitan Mexico City residents [45,46,47,48,49,50,51,52,53,54,55,56]. Significant caudate nuclei and cerebellar gray and white matter atrophy were also documented [57,58,59,60,61,62,63,64,65,66,67,68].

Strikingly, the poorer cognitive performance in MMC residents was associated with caudate and left orbital gyrus atrophy in keeping with orbital frontal cortex connections with regions processing visual, spatial, emotional information and social cognition, PFC-caudate brain wiring, and caudate functions affecting learning, memory, reward and motivation [69,70,71,72,73,74]. The current findings are not unexpected, i.e., the cerebellar atrophy is significant and could have a clinical counterpart in gait and equilibrium abnormalities described by our group in young MMC urbanites [75], cognitive deficits [16,57], the association between hearing loss and decreased brainstem and cerebellar volumes in AD cases [76], and gait and cognitive abnormalities associated with regional cerebellar atrophy in elderly fallers [77]. Cerebellar atrophy is particularly intriguing in view of our published cerebellar higher concentrations of magnetite (estimated from their saturation remanent magnetisation (SIRM) values) in MMC young forensic cases [2] and the recent description of regional cerebellar hypermetabolism in AD [78].

Hemispheric cortical significant differences documented in subjects ≤30 vs. ≥31 years old are of deep concern, as targeted cortical thickness and surface area regions involved overlap with those described in AD, PD, Lewy body disease (LBD) spectrum and FTD [45,46,47,48,51,52,53,54,79,80,81,82]. Brain MRI changes associated with early-onset Alzheimer’s disease (EOAD; aged < 65 years) (A + T + N +), in Contador et al., work [51] versus 19 controls (A-T-N-) found EOAD longitudinal atrophy spread with a posterior-to-anterior gradient with hippocampus/amygdala atrophy. In contrast, in MMC cases, decreased cortical thickness and surface area affected frontal and middle temporal regions and resulted in significant differences in global gray matter measurements (*p* =< 0.0001) and CSF (*p* = 0.0008) in ≤30 vs. ≥31 years old. Moreover, we did not document significant differences in the hippocampal or amygdala regions. This is remarkable, because MoCA LIS scores (animal naming, sentence repetition and word fluency) were already a key cognitive target (scores below the cut-off of 5.5, normal score 6) in MMC ≤ 30 years old, pointing to temporal-parietal-frontal circuit early and progressive involvement [83,84,85,86,87,88,89]. Language deficits are an early indicator in AD, and abnormal verbal task performance is an important diagnostic criterion for both AD and MCI [88].

MoCA index scores are valid measures in MCI, and Kim et al., [90] have shown MoCA-OIS (Orientation) and MoCA total score distinguished between MCI and dementia groups in both AD and vascular cognitive impairment (VCI). In MMC ≥ 31 years old, the combined score of delay recall, EIS, VIS and LIS reached values below the cutoff score. Indeed, LIS scores were strongly associated with decreased volumes in the right temporal superior transverse, left cingulate middle posterior and anterior gyrus and sulcus and the left orbital gyrus.

The targeted predominantly anterior brain atrophy is critical, since we have shown extensive cortical and brainstem progressive proteinopathies associated with AD, PD and TDP-43 pathology and neuroinflammation in 203 consecutive forensic autopsies of MMC ≤ 40 years old individuals, and clinically abnormal brainstem auditory evoked potentials (BAEPs), stress and sleep behaviour disorders, gait and equilibrium abnormalities, and CSF and brain MRS AD markers in pediatric and young adult ages [1,2,75,91,92,93,94,95,96,97].

In the background of 21.8 million MMC residents with sustained exposures to PM_2.5_ above USEPA annual standards, Falcon et al.’s work [55] is indeed an obligated reference. Falcon and coworkers [55] documented significant changes in cerebral volumes for Barcelona’s cognitively intact residents average age 58.6 years old with 80% family history of AD and ~50% APOE4 allele carriers. Specifically, exposures to NO_2_ were associated with decreased gray matter (GM) in the precuneus and greater WM volume in the splenium of the corpus callosum and inferior longitudinal fasciculus, while PM_2.5_ exposures above the USEPA annual standard (16.3 μg/m^3^ range 8.4–24.2) were associated with greater GM in the cerebellum and WM in the splenium of the corpus callosum, the superior longitudinal fasciculus, and cingulate gyrus. It is crucial to emphasize that Falcon’s study was done in cognitively intact individuals with significant AD genetic risk, while ours was done in subjects with average MoCA scores in the range of MCI, no genetic risk factors and lifetime exposures to PM_2.5_ concentrations significantly higher than in Barcelona.

The key issue in Falcon et al.’s work is the impact of specific air pollutants upon GM and WM volumes of targeted brain areas in a highly genetically vulnerable population even before cognitive changes take place. In sharp contrast, in MMC residents, the historically documented early neuropathological development of AD, PD and TDP-43 alterations is critical to the hemispheric and subcortical volume changes recorded for an adult middle-aged population that already has cognitive deficits.

The bilateral caudate atrophy (R > L) and their relationship to MoCA total scores deserves a comment. The caudate gray matter volume association with the degree of social intelligence (SI) as measured by the Guilford-Sullivan test was explored by Votinov and coworkers [98]. Their findings of the Guilford-Sullivan test positively correlating with the FC between seeds in the right caudate head and two clusters within the right superior temporal gyrus and bilateral precuneus are remarkable since both regions are part of the theory of mind (ToM) network. Social intelligence deficits [99,100], intense apathy [64] and poorer cognitive function are all related to caudate function and disruption of associative and limbic pathways from/to the PFC and are described in PD patients [62,63]. Interestingly, global measures of striatum such as total striatum, nucleus accumbens, caudate nuclei, and putamen may not be significantly different between PD patients and controls, although inward surface displacement of caudal-motor striatum (the region first and most dopamine depleted in PD) distinguished PD patients from controls in the work of Khan et al. [101]. The integrity of white matter cortico-striatal connections in caudal-motor and adjacent striatal sub-regions (i.e., executive and temporal striatum) was reduced for PD patients relative to controls [101]. Interestingly, loneliness and human emotions are related to changes to the ventral striatum and cerebellum [57,102], while in adolescents, negative associations were found between left hemisphere caudate volume and scores on ‘total wellbeing’ [103].

The issue of the significant caudate involvement in MMC residents is critical for many reasons: (1) We have described neuropathological early PD in 23% of forensic autopsies in MMC individuals ≤40 years old and brainstem, olfactory bulb and neuroenteric system α-synuclein starting in children and progressing as the individuals grow older in Mexico City [3,91,92,104,105]; (2) In the same forensic study, we have documented extensive hyperphosphorylated tau in substantia nigrae in adolescents [3]; and (3) The literature is very clear regarding the association between structural brain changes in rapid eye movement sleep disorders (RBD) and PD, and in fact RBD is considered a prodromal state of PD [58]. RBD individuals in the Holtbernd et al., study [58] showed increased volume of the right caudate nucleus compared to controls, and higher cerebellar volume compared with both PD subjects and controls.

In sharp contrast, PD patients had decreased volumes in the basal ganglia, midbrain, pedunculopontine nuclei, and cerebellum [58]. Remarkably, Holtbernd et al., commented upon the “co-occurrence of neurodegeneration and compensatory mechanisms that fail with emerging PD pathology” [58]. In our MMC middle-aged adults, caudate atrophy is already present and pRBD [94] is documented in 32.7% of MMC residents with PTSD, raising the issue of PTSD and neurodegeneration association in highly exposed air pollution residents [106,107,108,109] and the strong possibility of establishing a link between RBD documented by polysomnography and caudate atrophy in future MMC studies.

It is clear for MMC residents that a convergent pathophysiological hallmark of PD is affecting the striatum, as described in PD patients by Li et al. [65]. In their study of 84 PD patients versus 70 matched healthy controls, the modulation of early caudate atrophy over other brain structures showed that GM atrophy progressively expands from basal ganglia to the angular gyrus, temporal areas, and eventually spreads through the subcortical-cortical networks as PD progresses. Remarkably, Li et al. [65] identified a shared caudate-associated degeneration network including the basal ganglia, thalamus, cerebellum, sensorimotor cortex, and cortical association areas with the PD progressive factors. The authors emphasized the importance of the early caudate atrophy and potential clinical applications in the development of early predictors of PD onset and progress [65]. The striking caudate atrophy (and cerebellar involvement) in our MMC subjects in the presence of autonomic data in children and young adults (personal communication with Nora Vacaseydel MD) obligate us to consider such finding as an early future predictor of PD onset in highly exposed air pollution individuals [110,111,112]. The involvement of somatosensory cortices (in PD [65]) and decreased functional connectivity in the Default Mode Network (DMN) in AD as discussed by Weisman et al., and Becci and Giacomussi’s work [113,114] add significant knowledge to this discussion. Weismann et al. [113] commented that cognitive domains modulate cortical somatosensory processing and when attention and processing speed abilities are considered in AD cases, differences in gamma-frequency somatosensory response amplitude and gating become obvious and are accompanied by statistical suppression effects, thus early documentation of somatosensory processing is critical for identifying individuals at high risk of proteinopathies.

MMC middle-age adults are showing an MRI overlap in cortical and subcortical structures involved in AD, PD, LBD and FTD associated to cognitive decline and our deep concern relates to three main issues: (1) The need to identify subjects at risk as early as possible, and to predict the progression of the neurodegenerative processes at a stage when we can still effectively intervene; (2) The extensive hemispheric cortical and subcortical involvement in young urbanites is likely already impacting key processes such as social intelligence, the high risk for eating disorders, attention-deficit/hyperactivity disorder, conduct and sleep disorders, bullying behaviors, and depression [98,99,100,115,116,117]; and (3) The study of the progression of the structural brain changes and its association with clinical variables, including cognition deficits, should be of great concern for researchers studying highly exposed air pollution, children, teens and young adults.

The paradigm of neurodegenerative disease being associated with aging certainly does not apply in the setting of high air pollution scenarios, and thus we are obligated to develop guidance on the use of cognitive testing, neuroimaging and diagnosing potential early clinical neurodegenerative manifestations such as abnormal gait, BAEPS, PTSD and sleep disorders in young urbanites.

The current preclinical criteria aimed at elderly populations that is being used in the current literature [118,119,120,121,122,123] is certainly not ideal. We must agree with Dubois et al., [124] that the NIA-AA [119] that relies on biomarkers has limitations, and that those limitations certainly apply to young cohorts with no comorbidities, but with cognition deficits associated with extensive MRI changes. Specifically, we know that 55% of the MMC population (average age 21.6 ± 5.8 years, with 13.7 ± 1.3 formal education years) is already cognitively impaired, but we are very aware it will be extremely difficult to apply the ATN biomarker classification system (amyloid beta [A], pathologic tau [T], and neurodegeneration [N]) for predicting conversion from mild cognitive impairment (MCI) to dementia [118,119,120]. Moreover, of deep concern is the fact that in Aβ-positive elderly [121], 16% of variance in cross-sectional cognitive impairment was accounted for by Aβ, 46–47% by tau, and 25–29% by atrophy, and the Aβ-tau-atrophy pathway accounted for 50% to 56% of variance in longitudinal cognitive decline [121].

Furthermore, to complicate matters, current AD progression studies [122] support pivotal roles for regional amyloid beta (Aβ) and tau deposition, the identification of genetic contributions and comorbidities such as vascular disease that we do not have in children and young adults.

We are in the position of going to the ATX(N) system [123] applicable to children and young adults and incorporating novel biomarkers such as neuroimmune dysregulation and blood-brain barrier alterations that are already documented in children [2,3,13,14].

The study has several strengths, including the selection of clinically healthy college- educated individuals, with no family history of neurodegenerative diseases, no exposure to harmful drugs, including alcohol, or head injury of any etiology and with lifelong residency in MMC. Moreover, we performed the neuropathological studies of the 203 forensic MMC autopsies in a cohort with similar backgrounds as our MRI cases, which allowed us to establish associations with AD, PD and TDP-43 pathology discussed in the forensic cases [2,3]. Limitations should be noted. It is a small sample in a cross-sectional design limiting causal inference, and due to the COVID epidemic we were unable to include the planned controls for which we had the MoCA studies. However, our results remained robust in terms of the extensive hemispheric cortical thickness and surface volume involvement and the caudate and cerebellar atrophy in a population for which we had documented high concentrations of magnetite in the cerebellum, PD neuropathology in 23% of consecutive 203 MMC autopsies, gait and equilibrium abnormalities and sleep disorders [2,3,4,75,94].

## 5. Conclusions

Metropolitan Mexico City children and young adults historically have neuropathological hallmarks of AD, PD and TDP-43 pathology. The current study found MMC residents exposed to sustained, yearlong PM2.5 concentrations averaging 30.9 µg/m^3^ per year (well above the USEPA annual standard 12 µg/m^3^) showed robust hemispheric differences in frontal and temporal lobes, caudate and cerebellar gray and white matter when compared to ≤30 vs. ≥31 years old residents. Significant associations between MoCA total scores and caudate bilateral volumes and specific reductions in frontal and temporal cortical thickness and cerebellar white matter are remarkable in a healthy population with no comorbidities. Residency in Metropolitan Mexico City is associated with multi-domain cognitive impairment and an overlap of targeted patterns of structural brain atrophy as seen in AD, PD and FTD.

Prospective follow-up studies of MMC children and young adults are urgently needed to determine the impact on brain development and cognitive impairment and brain atrophy risk across pediatric and young adulthood ages.

PM2.5 emissions controls, including regulating heavy diesel vehicles, should be prioritized. We need an early biomarker identification of neurodevelopmental and neurodegenerative effects of air pollution in young MMC residents. This is a serious health crisis, and we desperately need support.

## Figures and Tables

**Table 1:** Summary of MoCA scores, age, BMI, formal education years and memory score in the MMC 233 subjects, the control city of Hermosillo 35 group versus the MMC MRI + MoCA 34 group.

Residency	MoCA Scores	Average Age Years	BMI	Education Years	Memory
MMC ≥ 31 years old n: 83	20.4 ± 3.4	46.4 ± 11.8	27.8 ± 3.9	13.2 ± 3.3	1.4 ± 1.4
MRI MMC ≥ 31 years old n: 14	23.3 ± 2.8	42.7 ± 9.3	28.1 ± 4.3	16.0 ± 2.1	2.0 ± 1.4
CONTROL ≥ 31 years old n: 13	25.2 ± 2.3	44.0 ± 7.2	26.9 ± 4.3	15.2 ± 2.8	3.3 ± 1.7
MMC ≤ 30 years old n: 150	24.2 ± 2.6	21.6 ± 3.5	24.2 ± 3.2	13.6 ± 1.7	2.7 ± 1.4
MRI MMC ≤ 30 years old n: 20	24.5 ± 2.6	22.0 ± 3.3	23.8 ± 3.7	14.5 ± 1.6	2.5 ± 1.2
CONTROL ≤ 30 years old n: 22	24.7 ± 2.1	19.3 ± 1.3	21.9 ± 2.7	13.7 ± 0.6	3.1 ± 1.3
ALL MRI MMC n: 34	23.9 ± 2.7	32.4 ± 6.3	25.9 ± 4.0	15.2 ± 1.8	2.25 ± 1.3
MMC n: 233	22.3 ± 3	33.95 ± 7.65	26 ± 3.55	13.4 ± 2.5	2.05 ± 1.4
CONTROL City n: 35	24.95 ± 2.2	31.65 ± 4.25	24.4 ± 3.5	14.45 ± 1.7	3.2 ± 1.5

**Table 2:** MRI volume corrected for Multiple Comparisons in MMC subjects: *p* values after correcting ICV linearly and FDR. All subjects are right-handed.

Anatomical Region	≤30 Years Mean SD	≥31 Years Mean SD	*p* Value Corrected FDR
**VOLUME DATA**			
WHITE MATTER	455,195 ± 40,045	447,456 ± 38,578	0.9285
GRAY MATTER	754,913 ± 46,840	715,865 ± 59,604	<0.0001
CSF	244,804 ± 26,320	270,899 ± 33,336	0.000851
LEFT CEREBELLUM WM	13,223 ± 1465	12,083 ± 1139	0.0715
RIGHT CEREBELLUM WM	13,101 ± 1408	11,762 ± 921	0.014468
LEFT CEREBELLUM CORTEX	48,482 ± 4679	42,265 ± 3688	0.000114
RIGHT CEREBELLUM CORTEX	49,084 ± 4538	42,353 ± 3409	<0.0001
LEFT CAUDATE	3529 ± 378	3216 ± 319	0.0153
RIGHT CAUDATE	3682 ± 382	3320 ± 328	0.006498
LEFT PUTAMEN	5115 ± 639	4610 ± 656	0.0993
RIGHT PUTAMEN	5004 ± 731	4561 ± 682	0.1785
LEFT PALLIDUM	1829 ± 368	1716 ± 222	0.6175
RIGHT PALLIDUM	1753 ± 323	1654 ± 269	0.5656
LEFT HIPPOCAMPUS	3986 ± 321	4011 ± 346	0.5777
RIGHT HIPPOCAMPUS	4162 ± 434	4166 ± 388	0.4395
LEFT AMYGDALA	1503 ± 163	1472 ± 155	0.8599
RIGHT AMYGDALA	1600 ± 190	1559 ± 168	0.8045
LEFT ACCUMBENS AREA	634 ± 105	555 ± 115	0.0377
RIGHT ACCUMBENS AREA	583 ± 99	517 ± 83	0.0758
OPTIC CHIASM	231 ± 33	256 ± 24	0.007
CORPUS CALLOSUM POST	982 ± 176	939 ± 126	0.6781
CORPUS CALLOSUM MIDPOSTERIOR	534 ± 110	468 ± 76	0.1625
CORPUS CALLOSUM CENTRAL	511 ± 103	441 ± 67	0.0981
**SURFACE AREA DATA**			
LEFT GYRUS FRONTAL INF OPERCULAR	3269 ± 491	2777 ± 426	0.013
RIGHT GYRUS FRONTAL INF OPERCULAR	3261 ± 475	2813 ± 490	0.0289
LEFT GYRUS FRONTAL INF ORBITAL	1193 ± 168	1057 ± 155	0.0385
LEFT GYRUS FRONTAL MIDDLE	9961 ± 931	8659 ± 1110	0.004605
LEFT GYRUS OCC TEMP MEDIAL LINGUAL	4653 ± 591	4095 ± 621	0.0397
LEFT GYRUS ORBITAL	5985 ± 702	5451 ± 787	0.0440
LEFT PARIETAL INF ANGULAR	5627 ± 970	4912 ± 871	0.0313
LEFT GYRUS TEMPORAL INFERIOR	7085 ± 1306	6307 ± 1085	0.0352
LEFT GYRUS TEMPORAL MIDDLE	7214 ± 757	6411 ± 627	0.000855
LEFT LAT FIS ANTERIOR HORIZONTAL	424 ± 86	323 ± 93	0.0082
LEFT LAT FIS ANTERIOR VERTICAL	480 ± 84	379 ± 85	0.002906
LEFT SULCUS ORBITAL LATERAL	513 ± 122	397 ± 104	0.005822
LEFT SULCUS ORBITAL H SHAPED	2453 ± 335	2149 ± 374	0.013217
RIGHT GYRI & SULCUS OCCIPITAL INFERIOR	2494 ± 364	2147 ± 500	0.0425
RIGHT GYRI & SULCUS CINGULATE ANTERIOR	5211 ± 456	4706 ± 749	0.015274
RIGHT G CUNNEUS	2984 ± 405	2692 ± 281	0.0675
RIGHT G FRONTAL INF OPERCULAR	3261 ± 475	2813 ± 490	0.0289
RIGHT G OCCIPITAL TEMP LAT FUSIFORM	4761 ± 592	4208 ± 661	0.014617
RIGHT G PRECENTRAL	6477 ± 794	5707 ± 995	0.0316
RIGHT G TEMP SUP GT TRANSVERSE	778 ± 115	677 ± 105	0.0349
RIGHT G TEMP SUPERIOR LATERAL	4754 ± 527	4329 ± 459	0.0320
RIGHT G TEMPORAL INFERIOR	6768 ± 1074	5917 ± 964	0.0255
RIGHT G TEMPORAL MIDDLE	8176 ± 892	7175 ± 864	0.00021
RIGHT LAT ANT FIS HORIZONTAL	531 ± 173	425 ± 114	0.0488
RIGHT SULCUS OCCIP MIDDLE LUNATUS	1191 ± 288	999 ± 175	0.0588
RIGHT SULCUS orbital MED OLFACTORY	972 ± 125	899 ± 111	0.0291
RIGHT SULCUS H SHAPED	2375 ± 339	2099 ± 249	0.013
RIGHT SULCUS TEMPORAL SUPERIOR	8880 ± 1142	8038 ± 898	0.0498
LEFT G PARIETAL ING ANGULAR	5628 ± 971	4913 ± 871	0.0313
LEFT G TEMPORAL MIDDLE	7215 ± 758	6412 ± 628	0.0009
**CORTICAL THICKNESS DATA**			
LEFT G&S SUBCENTRAL	2.69 ± 0.17	2.55 ± 0.14	0.02358
LEFT G&S TRANV FRONTAL POL	2.71 ± 0.17	2.55 ± 0.13	0.00318
LEFT G&S CINGULAR ANTERIOR	2.66 ± 0.15	2.49 ± 0.15	0.004167
LEFT G&S CINGULATE MID ANTERIOR	2.68 ± 0.19	2.47 ± 0.19	0.008505
LEFT G&S CINGULATE MID POSTERIOR	2.57 ± 0.17	2.33 ± 0.33	<0.0001
LEFT FRONTAL INF OPERCULAR	2.77 ± 0.15	2.61 ± 0.15	0.008622
LEFT G FRONTAL INF TRIANGULAR	2.69 ± 0.17	2.54 ± 0.14	0.00665
LEFT FRONTAL MIDDLE	2.64 ± 0.14	2.53 ± 0.09	0.01592
LEFT G FRONTAL SUPERIOR	2.98 ± 0.14	2.81 ± 0.12	0.00036
LEFT G OCCIPITAL TEMP LAT FUSIFORM	2.91 ± 0.15	2.81 ± 0.14	0.01941
LEFT G OCC TEMP MEDIAL LINGUAL	2.06 ± 0.07	1.95 ± 0.08	0.00075
LEFT G PRECUNNEUS	2.43 ± 0.12	2.32 ± 0.17	0.0292
LEFT LAT FIS ANTERIOR HORIZONTAL	2.34 ± 0.28	2.09 ± 0.22	0.0257
LEFT LAT FIS ANTERIOR VERTICAL	2.30 ± 0.34	2.02 ± 0.21	0.01803
LEFT S CALCARINE	1.89 ± 0.12	1.80 ± 0.095	0.0544
LEFT S CINGULAR INSULA SUPERIOR	2.57 ± 0.12	2.43 ± 0.14	0.00451
LEFT S FRONTAL SUPERIOR	2.44 ± 0.16	2.32 ± 0.13	0.0068
LEFT S PRECENTRAL SUPERIOR PAR	2.42 ± 0.11	2.28 ± 0.21	0.01671
LEFT G&S CINGULATE ANTERIOR	2.58 ± 0.17	2.43 ± 0.10	0.0097
LEFT G&S CINGULATE MID ANTERIOR	2.66 ± 0.14	2.50 ± 0.13	0.0053
LEFT G&S CINGULATE MID POSTERIOR	2.58 ± 0.12	2.43 ± 0.13	0.0020
LEFT G CINGULATE POSTCENTRAL	2.87 ± 0.24	2.68 ± 0.15	0.01919
LEFT G CUNNEUS	1.85 ± 0.11	1.75 ± 0.07	0.01230
LEFT G FRONTAL INF OPERCULAR	2.78 ± 0.18	2.59 ± 0.21	0.0245
LEFT G FRONTAL INF TRIANGULAR	2.76 ± 0.17	2.60 ± 0.18	0.0147
LEFT G FRONTAL MIDDLE	2.65 ± 0.11	2.55 ± 0.11	0.0172
LEFT G FRONTAL SUPERIOR	2.95 ± 0.14	2.79 ± 0.1	0.0016
LEFT OCCIPITAL MIDDLE	2.63 ± 0.12	2.55 ± 0.08	0.0319
LEFT SUP TEMP LATERAL FUSIFORM	2.99 ± 0.14	2.86 ± 0.16	0.0137
LEFT G PARIETAL INF SUPRAMARGINAL	2.73 ± 0.11	2.64 ± 0.13	0.0386
LEFT G PRECENTRAL	2.96 ± 0.14	2.78 ± 0.28	0.0103
LEFT G CUNNEUS	2.43 ± 0.13	2.34 ± 0.15	0.0676
LEFT CIRCULAR INSULAR SUPERIOR	2.61 ± 0.10	2.47 ± 0.16	0.011
LEFT S FRONTAL SUPERIOR	2.42 ± 0.16	2.28 ± 0.14	0.016
LEFT S PRECENTRAL SUPERIOR	2.44 ± 0.17	2.30 ± 0.22	0.026

**Table 3:** The adjusted *p* values (adjusted for age and intracranial volume) for the significance of the linear association between MoCA Index scores and regional cerebral volume.

**MoCA total Score**	
Right Caudate	0.0065
Left Caudate	0.009
Left gyrus orbital	0.0014
**Orientation**	
Right Gyrus temporal sup transverse	0.0045
**EIS**	
Right cerebellar white matter	0.029
Left sulcus orbital lateral	0.0019
Left gyrus frontal inferior triangular	0.023
**LIS**	
Left gyrus orbital	0.026
Right gyrus temporal superior transverse	0.0071
Right gyrus temporal inferior	0.026
Left gyrus and sulcus cingulate middle posterior	0.0084
Left gyrus and sulcus cingulate middle anterior	0.021
**VIS**	
Left gyrus orbital	0.0184
Left sulcus orbital lateral	0.0030
**AIS**	
Right gyrus temporal superior transverse	0.0063
**SUMMARY SCORE**	
Left gyrus orbital	0.0009
Left sulcus orbital lateral	0.021
Left gyrus and sulcus subcentral	0.023
Right gyrus temporal superior lateral	0.028

## Data Availability

Data supporting reported results are included in this work.

## Supplementary Materials

The following supporting information can be downloaded at: https://www.mdpi.com/article/10.3390/toxics10040156/s1, Table S1: Summary of MoCA cognition scores in subjects ≥ 31 y and ≤30 y in Metropolitan Mexico City MRI and non-MRI subjects and Control City Hermosillo. Ad-justed p-value refers to the p-values after removing the linear effects of age, gender, BMI, and education years. and Table S2: Summary of MoCA scores, Cognitive Domain Scores, age, BMI and education years in MMC (MRI and NON-MRI) and Control City Hermosillo ≤ 30 y and ≥31 y. Adjusted *p*-value refers to the *p*-values after removing the linear effects of age, gender, BMI, and education years.

## References

[1] Calderón-Garcidueñas L., Pérez-Calatayud A.A., González-Maciel A., Reynoso-Robles R., Silva-Pereyra H.G., Ramos-Morales A., Torres-Jardón R., Soberanes-Cerino C.D.J., Carrillo-Esper R., Briones-Garduño J.C. (2022). Environmental Nanoparticles Reach Human Fetal Brains. Biomedicines.

[2] Calderón-Garcidueñas L., Gónzalez-Maciel A., Reynoso-Robles R., Delgado-Chávez R., Mukherjee P.S., Kulesza R.J., Torres-Jardón R., Ávila-Ramírez J., Villarreal-Ríos R. (2018). Hallmarks of Alzheimer disease are evolving relentlessly in Metropolitan Mexico City infants, children and young adults. APOE4 carriers have higher suicide risk and higher odds of reaching NFT stage V at ≤ 40 years of age. Environ Res..

[3] Calderón-Garcidueñas L., González-Maciel A., Reynoso-Robles R., Hammond J., Kulesza R., Lachmann I., Torres-Jardón R., Mukherjee P.S., Maher B.A. (2020). Quadruple abnormal protein aggregates in brainstem pathology and exogenous metal-rich magnetic nanoparticles (and engineered Ti-rich nanorods). The substantia nigrae is a very early target in young urbanites and the gastrointestinal tract a key brainstem portal. Environ. Res..

[4] Maher B.A., Ahmed I.A.M., Karloukovski V., MacLaren D.A., Foulds P.G., Allsop D., Mann D.M.A., Torres-Jardón R., Calderon-Garciduenas L. (2016). Magnetite pollution nanoparticles in the human brain. Proc. Natl. Acad. Sci. USA.

[5] Jung C.R., Lin Y.T., Hwang B.F. (2015). Ozone, particulate matter, and newly diagnosed Alzheimer’s disease: A population-based cohort study in Taiwan. J. Alzheimer’s Dis..

[6] Chen H., Kwong J.C., Copes R., Tu K., Villeneuve P., van Donkelaar A., Hystad P., Martin R.V., Murray B., Jessiman B. (2017). Living near major roads and the incidence of dementia, Parkinson’s disease, and multiple sclerosis: A population-based cohort study. Lancet.

[7] Russ T.C., Cherrie M.P., Dibben C., Tomlinson S., Reis S., Dragosits U., Vieno M., Beck R., Carnell E., Shortt N.K. (2021). Life Course Air Pollution Exposure and Cognitive Decline: Modelled Historical Air Pollution Data and the Lothian Birth Cohort 1936. J. Alzheimer’s Dis..

[8] Mortamais M., Gutierrez L.-A., de Hoogh K., Chen J., Vienneau D., Carrière I., Letellier N., Helmer C., Gabelle A., Mura T. (2021). Long-term exposure to ambient air pollution and risk of dementia: Results of the prospective Three-City Study. Environ. Int..

[9] Grande G., Ljungman P.L.S., Eneroth K., Bellander T., Rizzuto D. (2020). Association Between Cardiovascular Disease and Long-term Exposure to Air Pollution with the Risk of Dementia. JAMA Neurol..

[10] Tham R., Schikowski T. (2021). The Role of Traffic-Related Air Pollution on Neurodegenerative Diseases in Older People: An Epidemiological Perspective. J. Alzheimer’s Dis..

[11] Porta D., Narduzzi S., Badaloni C., Bucci S., Cesaroni G., Colelli V., Davoli M., Sunyer J., Zirro E., Schwartz J. (2016). Air pollution and cognitive development at age seven in a prospective Italian birth cohort. Epidemiology.

[12] Kicinski M., Vermeir G., Van Larebeke N., Hond E.D., Schoeters G., Bruckers L., Sioen I., Bijnens E., Roels H.A., Baeyens W. (2015). Neurobehavioral performance in adolescents is inversely associated with traffic exposure. Environ. Int..

[13] Calderón-Garcidueñas L., Mora-Tiscareño A., Ontiveros E., Gómez-Garza G., Barragán-Mejía G., Broadway J., Chapman S., Valencia-Salazar G., Jewells V., Maronpot R.R. (2008). Air pollution, cognitive deficits and brain abnormalities: A pilot study with children and dogs. Brain Cogn..

[14] Calderón-Garcidueñas L., Engle R., Mora-Tiscareño A., Styner M., Gómez-Garza G., Zhu H., Jewells V., Torres-Jardón R., Romero L., Monroy-Acosta M.E. (2011). Exposure to severe urban air pollution influences cognitive outcomes, brain volume and systemic inflammation in clinically healthy children. Brain Cogn..

[15] Calderón-Garcidueñas L., Torres-Jardón R., Kulesza R.J., Mansour Y., González-González L.O., Gónzalez-Maciel A., Reynoso-Robles R., Mukherjee P.S. (2020). Alzheimer disease starts in childhood in polluted Metropolitan Mexico City. A major health crisis in progress. Environ. Res..

[16] Calderón-Garcidueñas L., Mukherjee P.S., Kulesza R.J., Torres-Jardón R., Hernández-Luna J., Ávila-Cervantes R., Macías-Escobedo E., González-González O., González-Maciel A., García-Hernández K. (2019). Mild Cognitive Impairment and Dementia In-volving Multiple Cognitive Domains in Mexican Urbanites. J. Alzheimer’s Dis..

[17] Calderón-Garcidueñas L., Chávez-Franco D.A., Luévano-Castro S.C., Macías-Escobedo E., Hernández-Castillo A., Carlos-Hernández E., Franco-Ortíz A., Castro-Romero S.P., Cortés-Flores M., Crespo-Cortés C.N. (2022). Metals, Nanoparticles, Particulate Matter, and Cognitive Decline. Front. Neurol..

[18] Dale A.M., Fischl B., Sereno M.I. (1999). Cortical surface-based analysis. I. Segmentation and surface reconstruction. NeuroImage.

[19] Hagler D.J., Hatton S., Cornejo M.D., Makowski C., Fair D.A., Dick A.S., Sutherland M.T., Casey B., Barch D.M., Harms M.P. (2019). Image processing and analysis methods for the Adolescent Brain Cognitive Development Study. NeuroImage.

[20] Secretaría del Medio Ambiente de la Ciudad de México (2021). http://www.aire.cdmx.gob.mx/default.php.

[21] Nasreddine Z.S., Phillips N.A., Bédirian V., Charbonneau S., Whitehead V., Collin I., Cummings J.L., Chertkow H. (2005). The Montreal Cognitive Assessment, MoCA: A Brief Screening Tool for Mild Cognitive Impairment. J. Am. Geriatr. Soc..

[22] Julayanont P., Brousseau M., Chertkow H., Phillips N., Nasreddine Z.S. (2014). Montreal cognitive assessment memory index score (MoCA-MIS) as a predictor of conversion from mild cognitive impairment to Alzheimer’s disease. J. Am. Geriatr. Soc..

[23] Pugh E.A., Kemp E.C., van Dyck C.H., Mecca A.P., Sharp E.S. (2018). Effects of Normative Adjustments to the Montreal Cognitive Assessment. Am. J. Geriatr. Psychiatry.

[24] Loureiro C., Garcia C., Adana L., Yacelga T., Rodriguez-Lorenzana A., Maruta C. (2018). Use of the Montreal Cognitive Assessment (MoCA) in Latin America: A systematic review. Rev. De Neurol..

[25] (2013). Diagnostic and Statistical Manual of Mental Disorders.

[26] Petersen R.C. (2004). Mild cognitive impairment as a diagnostic entity. J. Intern. Med..

[27] Torres-Jardón R., Núñez G.S. (2018). Politicas públicas y su efecto en la calidad del aire de la Zona metropolitana de la Ciudad de Mexico. Transversalidad de la Politica del Aire en Mexico.

[28] Velasco E., Retama A. (2017). Ozone’s threat hits back Mexico City. Sustain. Cities Soc..

[29] Zavala M., Brune W.H., Velasco E., Retama A., Cruz-Alvarez L.A., Molina L.T. (2020). Changes in ozone production and VOC reac-tivity in the atmosphere of the Mexico City Metropolitan Area. Atmos. Environ..

[30] Molina L.T., Velasco E., Retama A., Zavala M. (2019). Experience from Integrated Air Quality Management in the Mexico City Metropolitan Area and Singapore. Atmosphere.

[31] Dzepina K., Arey J., Marr L.C., Worsnop D.R., Salcedo D., Zhang Q., Onasch T.B., Molina L.T., Molina M.J., Jimenez J.L. (2007). Detection of particle-phase polycyclic aromatic hydrocarbons in Mexico City using an aerosol mass spectrometer. Int. J. Mass Spectrom..

[32] Mugica V., Mugica F., Torres M., Figueroa J. (2008). PM2.5Emission Elemental Composition from Diverse Combustion Sources in the Metropolitan Area of Mexico City. Sci. World J..

[33] Zavala M., Molina L.T., Yacovitch T.I., Fortner E.C., Roscioli J.R., Floerchinger C., Herndon S.C., Kolb C.E., Knighton W.B., Paramo V.H. (2017). Emission factors of black carbon and co-pollutants from diesel vehicles in Mexico City Atmos. Chem. Phys..

[34] Ladino L.A., Raga G.B., Baumgardner D. (2018). On particle-bound polycyclic aromatic hydrocarbons (PPAH) and links to gaseous emissions in Mexico city. Atmos. Environ..

[35] Amador-Muñoz O., Martínez-Domínguez Y., Gómez-Arroyo S., Peralta O. (2019). Current situation of polycyclic aromatic hydrocarbons (PAH) in PM2.5 in a receptor site in Mexico City and estimation of carcinogenic PAH by combining non-real-time and real-time measurement techniques. Sci. Total Environ..

[36] Adachi K., Buseck P.R. (2010). Hosted and Free-Floating Metal-Bearing Atmospheric Nanoparticles in Mexico City. Environ. Sci. Technol..

[37] Caudillo L., Salcedo D., Peralta O., Castro T., Alvarez-Ospina H. (2019). Nanoparticle size distributions in Mexico city. Atmospheric Pollut. Res..

[38] Velasco E., Retama A., Segovia E., Ramos R. (2019). Particle exposure and inhaled dose while commuting by public transport in Mexico City. Atmos. Environ..

[39] Múgica-Alvarez V., Figueroa-Lara J., Romero-Romo M., Sepulveda-Sanchez J., Lopez-Moreno T. (2012). Concentrations and pro-perties of airborne particles in the Mexico City subway system. Atmos. Environ..

[40] CCA (2014). Contaminación Ambiental en Hermosillo II: Expediente de Hechos Relativo a la Petición SEM-05-003.

[41] Del Río-Salas R., Ruiz J., De la O-Villanueva M., Valencia-Moreno M., Moreno-Rodríguez V., Gómez-Alvarez A., Grijalva T., Mendivil H., Paz-Moreno F., Meza-Figueroa D. (2012). Tracing geogenic and anthropogenic sources in urban dusts: Insights from lead isotopes. Atmos. Environ..

[42] Cruz-Campas M.E., Gomez-Alvarez A., Quintero-Nuñez M., Varela-Salazar J. (2013). One year air quality evaluation regarding total suspended particles (TSP) and heavy metals (Pb, Cd, Ni, Cu, Cr) in Hermosillo, Sonora, Mexico. Rev. Int. Contam. Ambient..

[43] Meza-Figueroa D., González-Grijalva B., Romero F., Ruiz J., Pedroza-Montero M., Rivero C.I.-D., Acosta-Elías M., Ochoa-Landin L., Navarro-Espinoza S. (2018). Source apportionment and environmental fate of lead chromates in atmospheric dust in arid environments. Sci. Total Environ..

[44] Rangel-López C.J. (2015). Diagnostic of the Origin and State of the Air Pollution in Hermosillo, Sonora.

[45] Iannopollo E., Garcia K., Alzheimer’s Disease Neuroimaging Initiative (2021). Enhanced detection of cortical atrophy in Alzheimer’s disease using structural MRI with anatomically constrained longitudinal registration. Hum. Brain Mapp..

[46] Pereira J.B., Svenningsson P., Weintraub D., Brønnick K., Lebedev A., Westman E., Aarsland D. (2014). Initial cognitive decline is as-sociated with cortical thinning in early Parkinson disease. Neurology.

[47] Korthauer L.E., Blujus J.K., Awe E., Frahmand M., Prost R., Driscoll I. (2021). Brain-behavior investigation of potential cognitive markers of Alzheimer’s disease in middle age: A multi-modal imaging study. Brain Imaging Behav..

[48] Contador J., Pérez-Millan A., Guillen N., Tort-Merino A., Balasa M., Falgàs N., Olives J., Castellví M., Borrego-Écija S., Bosch B. (2021). Baseline MRI atrophy predicts 2-year cognitive outcomes in early-onset Alzheimer’s disease. J. Neurol..

[49] Koenig L.N., LaMontagne P., Glasser M.F., Bateman R., Holtzman D., Yakushev I., Chhatwal J., Day G.S., Jack C., Mummery C. (2021). Regional age-related atrophy after screening for preclinical Alzheimer disease. Neurobiol. Aging.

[50] Kuhn T., Becerra S., Duncan J., Spivak N., Dang B.H., Habelhah B., Mahdavi K.D., Mamoun M., Whitney M., Pereles F.S. (2021). Translating state-of-the-art brain magnetic resonance imaging (MRI) techniques into clinical practice: Multimodal MRI differentiates dementia subtypes in a traditional clinical setting. Quant. Imaging Med. Surg..

[51] Contador J., Pérez-Millán A., Tort-Merino A., Balasa M., Falgàs N., Olives J., Castellví M., Borrego-Écija S., Bosch B., Fernández-Villullas G. (2021). Longitudinal brain atrophy and CSF biomarkers in early-onset Alzheimer’s disease. NeuroImage Clin..

[52] Platero C., Tobar M.C., Alzheimer’s Disease Neuroimaging Initiative (2020). Predicting Alzheimer’s conversion in mild cognitive impairment patients using longitudinal neuroimaging and clinical markers. Brain Imaging Behav..

[53] E Williams M., A Elman J., McEvoy L.K., A Andreassen O., Dale A.M., Eglit G.M.L., Eyler L.T., Fennema-Notestine C., E Franz C., A Gillespie N. (2021). 12-year prediction of mild cognitive impairment aided by Alzheimer’s brain signatures at mean age 56. Brain Commun..

[54] Amini M., Pedram M.M., Moradi A., Jamshidi M., Ouchani M. (2021). Single and Combined Neuroimaging Techniques for Alzheimer’s Disease Detection. Comput. Intell. Neurosci..

[55] Falcón C., Gascon M., Molinuevo J.L., Operto G., Cirach M., Gotsens X., Fauria K., Arenaza-Urquijo E.M., Pujol J., Sunyer J. (2021). Brain correlates of urban environmental exposures in cognitively unimpaired individuals at increased risk for Alzheimer’s disease: A study on Barcelona’s population. Alzheimer’s Dementia Diagn. Assess. Dis. Monit..

[56] Ingala S., De Boer C., A Masselink L., Vergari I., Lorenzini L., Blennow K., Chételat G., Di Perri C., Ewers M., van der Flier W.M. (2021). Application of the ATN classification scheme in a population without dementia: Findings from the EPAD cohort. Alzheimer’s Dement..

[57] Pierce J.E., Péron J. (2020). The basal ganglia and the cerebellum in human emotion. Soc. Cogn. Affect. Neurosci..

[58] Holtbernd F., Romanzetti S., Oertel W.H., Knake S., Sittig E., Heidbreder A., Maier A., Krahe J., Wojtala J., Dogan I. (2021). Convergent patterns of structural brain changes in rapid eye movement sleep behavior disorder and Parkinson’s disease on behalf of the German rapid eye movement sleep behavior disorder study group. Sleep.

[59] Devignes Q., Viard R., Betrouni N., Carey G., Kuchcinski G., Defebvre L., Leentjens A.F.G., Lopes R., Dujardin K. (2021). Posterior Cortical Cognitive Deficits Are Associated with Structural Brain Alterations in Mild Cognitive Impairment in Parkinson’s Disease. Front. Aging Neurosci..

[60] Crowley S.J., Banan G., Amin M., Tanner J.J., Hizel L., Nguyen P., Brumback B., Rodriguez K., McFarland N., Bowers D. (2021). Statistically Defined Parkinson’s Disease Executive and Memory Cognitive Phenotypes: Demographic, Behavioral, and Structural Neuroimaging Comparisons. J. Park. Dis..

[61] Shahmaei V., Faeghi F., Mohammadbeigi A., Hashemi H., Ashrafi F. (2019). Evaluation of iron deposition in brain basal ganglia of patients with Parkinson’s disease using quantitative susceptibility mapping. Eur. J. Radiol. Open.

[62] Camarda C., Torelli P., Pipia C., Battaglini I., Azzarello D., Rosano R., Ventimiglia C.C., Sottile G., Cilluffo G., Camarda R. (2019). Mild Parkinsonian Signs in a Hospital-based Cohort of Mild Cognitive Impairment Types: A Cross-sectional Study. Curr. Alzheimer Res..

[63] Owens-Walton C., Jakabek D., Li X., Wilkes F.A., Walterfang M., Velakoulis D., van Westen D., Looi J., Hansson O. (2018). Striatal changes in Parkinson disease: An investigation of morphology, functional connectivity and their relationship to clinical symptoms. Psychiatry Res. Neuroimaging.

[64] Levy R., Czernecki V. (2006). Apathy and the basal ganglia. J. Neurol..

[65] Li R., Zou T., Wang X., Wang H., Hu X., Xie F., Meng L., Chen H. (2021). Basal ganglia atrophy–associated causal structural network degeneration in Parkinson’s disease. Hum. Brain Mapp..

[66] Van den Berg K.R.E., Helmich R.C. (2021). The Role of the Cerebellum in Tremor—Evidence from Neuroimaging. Tremor Other Hyperkinetic Mov..

[67] Bede P., Chipika R.H., Christidi F., Hengeveld J.C., Karavasilis E., Argyropoulos G.D., Lope J., Shing S.L.H., Velonakis G., Dupuis L. (2021). Genotype-associated cerebellar profiles in ALS: Focal cerebellar pathology and cerebro-cerebellar connectivity alterations. J. Neurol. Neurosurg. Psychiatry.

[68] McKenna M.C., Chipika R.H., Shing S.L.H., Christidi F., Lope J., Doherty M.A., Hengeveld J.C., Vajda A., McLaughlin R.L., Hardiman O. (2021). Infratentorial pathology in frontotemporal dementia: Cerebellar grey and white matter alterations in FTD phenotypes. J. Neurol..

[69] Nestor P.G., Nakamura M., Niznikiewicz M., Thompson E., Levitt J.J., Choate V., Shenton M.E., McCarley R. (2013). In search of the functional neuroanatomy of sociality: MRI subdivisions of orbital frontal cortex and social cognition. Soc. Cogn. Affect. Neurosci..

[70] Jarbo K., Verstynen T.D. (2015). Converging Structural and Functional Connectivity of Orbitofrontal, Dorsolateral Prefrontal, and Posterior Parietal Cortex in the Human Striatum. J. Neurosci..

[71] Nestor P.G., Forte M., Ohtani T., Levitt J.J., Newell D.T., Shenton M.E., Niznikiewicz M., McCarley R.W. (2020). Faulty Executive Attention and Memory Interactions in Schizophrenia: Prefrontal Gray Matter Volume and Neuropsychological Impairment. Clin. EEG Neurosci..

[72] Levitt J.J., Zhang F., Vangel M., Nestor P.G., Rathi Y., Kubicki M., E Shenton M., O’Donnell L.J. (2021). The Organization of Frontostriatal Brain Wiring in Healthy Subjects Using a Novel Diffusion Imaging Fiber Cluster Analysis. Cereb. Cortex.

[73] Fariña A., Rojek-Giffin M., Gross J., De Dreu C.K.W. (2021). Social preferences correlate with cortical thickness of the orbito-frontal cortex. Soc. Cogn. Affect. Neurosci..

[74] Burks J.D., Conner A.K., Bonney P.A., Glenn C.A., Baker C.M., Boettcher L.B., Briggs R.G., O’Donoghue D.L., Wu D.H., Sughrue M.E. (2018). Anatomy and white matter connections of the orbitofrontal gyrus. J. Neurosurg..

[75] Calderón-Garcidueñas L., Torres-Solorio A.K., Kulesza R.J., Torres-Jardón R., González-González L.O., García-Arreola B., Chávez-Franco D.A., Luévano-Castro S.C., Hernández-Castillo A., Carlos-Hernández E. (2020). Gait and balance disturbances are common in young urbanites and associated with cognitive impairment. Air pollution and the historical development of Alzheimer’s disease in the young. Environ. Res..

[76] Llano D.A., Kwok S.S., Devanarayan V., Alzheimer’s Disease Neuroimaging Initiative (ADNI) (2021). Reported Hearing Loss in Alzheimer’s Disease Is Associated with Loss of Brainstem and Cerebellar Volume. Front. Hum. Neurosci..

[77] Droby A., El Mendili M.M., Giladi N., Hausdorff J.M., Maidan I., Mirelman A. (2021). Gait and cognitive abnormalities are associated with regional cerebellar atrophy in elderly fallers—A pilot study. Gait Posture.

[78] Gupta V., Booth S., Ko J.H. (2021). Hypermetabolic Cerebellar Connectome in Alzheimer’s Disease. Brain Connect..

[79] Scamarcia P.G., Agosta F., Caso F., Filippi M. (2021). Update on neuroimaging in non-Alzheimer’s disease dementia: A focus on the Lewy body disease spectrum. Curr. Opin. Neurol..

[80] Sarasso E., Agosta F., Piramide N., Filippi M. (2021). Progression of grey and white matter brain damage in Parkinson’s disease: A critical review of structural MRI literature. J. Neurol..

[81] Stage E.C., Svaldi D., Phillips M., Canela V.H., Duran T., Goukasian N., Risacher S.L., Saykin A.J., Apostolova L.G., Alzheimer’s Disease Neuroimaging Initiative (2020). Neurodegenerative changes in early- and late-onset cognitive impairment with and without brain amyloidosis. Alzheimer’s Res. Ther..

[82] Perry D., A Brown J., Possin K.L., Datta S., Trujillo A., Radke A., Karydas A., Kornak J., Sias A.C., Rabinovici G.D. (2017). Clinicopathological correlations in behavioural variant frontotemporal dementia. Brain.

[83] Okada T., Tanaka S., Nakai T., Nishizawa S., Inui T., Sadato N., Yonekura Y., Konishi J. (2000). Naming of animals and tools: A functional magnetic resonance imaging study of categorical differences in the human brain areas commonly used for naming visually presented objects. Neurosci. Lett..

[84] Bai H.M., Jiang T., Wang W.M., Li T.D., Liu Y., Lu Y.C. (2011). Functional MRI mapping of category-specific sites associated with naming of famous faces, animals and man-made objects. Neurosci. Bull..

[85] Meyers J.E., Volkert K., Diep A. (2000). Sentence Repetition Test: Updated Norms and Clinical Utility. Appl. Neuropsychol..

[86] Small J.A., Kemper S., Lyons K. (2000). Sentence repetition and processing resources in Alzheimer’s disease. Brain Lang..

[87] Beales A., Whitworth A., Cartwright J., Panegyres P.K., Kane R.T. (2019). Profiling sentence repetition deficits in primary progressive aphasia and Alzheimer’s disease: Error patterns and association with digit span. Brain Lang..

[88] Taler V., Phillips N.A. (2008). Language performance in Alzheimer’s disease and mild cognitive impairment: A comparative review. J. Clin. Exp. Neuropsychol..

[89] Mueller K.D., Hermann B., Mecollari J., Turkstra L.S. (2018). Connected speech and language in mild cognitive impairment and Alzheimer’s disease: A review of picture description tasks. J. Clin. Exp. Neuropsychol..

[90] Kim H., Yu K.-H., Lee B.-C., Kim B.-C., Kang Y. (2021). Validity of the Montreal Cognitive Assessment (MoCA) Index Scores: A Comparison with the Cognitive Domain Scores of the Seoul Neuropsychological Screening Battery (SNSB). Dement. Neurocogn. Disord..

[91] Calderón-Garcidueñas L., D’Angiulli A., Kulesza R.J., Torres-Jardón R., Osnaya N., Romero L., Keefe S., Herritt L., Brooks D.M., Avila-Ramirez J. (2011). Air pollution is associated with brainstem auditory nuclei pathology and delayed brainstem auditory evoked potentials. Int. J. Dev. Neurosci..

[92] Calderón-Garcidueñas L., González-González L.O., Kulesza R.J., Fech T.M., Pérez-Guillé G., Luna M.A.J.-B., Soriano-Rosales R.E., Solorio E., Miramontes-Higuera J.D.J., Chew A.G.-M. (2017). Exposures to fine particulate matter (PM2.5) and ozone above USA standards are associated with auditory brainstem dysmorphology and abnormal auditory brainstem evoked potentials in healthy young dogs. Environ. Res..

[93] Calderón-Garcidueñas L., Kulesza R.J., Mansour Y., Aiello-Mora M., Mukherjee P.S., González-González L.O. (2019). Increased Gain in the Auditory Pathway, Alzheimer’s Disease Continuum, and Air Pollution: Peripheral and Central Auditory System Dysfunction Evolves Across Pediatric and Adult Urbanites. J. Alzheimer’s Dis..

[94] Calderón-Garcidueñas L., Rajkumar R., Stommel E., Kulesza R., Mansour Y., Rico-Villanueva A., Flores-Vázquez J., Brito-Aguilar R., Ramírez-Sánchez S., García-Alonso G. (2021). Brainstem Quadruple Aberrant Hyperphosphorylated Tau, Beta-Amyloid, Alpha-Synuclein and TDP-43 Pathology, Stress and Sleep Behavior Disorders. Int. J. Environ. Res. Public Health.

[95] Calderón-Garcidueñas L., Ávila-Ramírez J., Calderón-Garcidueñas A., González-Heredia T., Acuna-Ayala H., Chao C.K., Thompson C., Ruiz-Ramos R., Cortés-González V., Martinez-Martinez L. (2016). Cerebrospinal Fluid Biomarkers in Highly Ex-posed PM 2∙5 Urbanites: The Risk of Alzheimer’s and Parkinson’s Diseases in Young Mexico City Residents. J. Alzheimer’s Dis..

[96] Calderón-Garcidueñas L., Mora-Tiscareño A., Melo-Sánchez G., Rodriguez-Diaz J., Torres-Jardon R., Styner M., Mukherjee P.S., Lin W., Jewells V. (2015). A critical Proton MR Spectroscopy marker of Alz-heimer’s disease early neurodegenerative change: Low hippocampal NAA/Cr ratio impacts APOE ɛ4 Mexico City children and their parents. J. Alzheimer’s Dis..

[97] Calderón-Garcidueñas L., Mora-Tiscareño A., Franco-Lira M., Zhu H., Lu Z., Solorio E., Torres-Jardón R., D’Angiulli A. (2015). Decreases in short term memory, IQ and altered brain metabolic ratios in urban apolipoprotein ε4 children exposed to air pollution. APOE modulates children’s brain air pollution responses. J. Alzheimer’s Dis..

[98] Votinov M., Myznikov A., Zheltyakova M., Masharipov R., Korotkov A., Cherednichenko D., Habel U., Kireev M. (2021). The Interaction between Caudate Nucleus and Regions Within the Theory of Mind Network as a Neural Basis for Social Intelligence. Front. Neural Circuits.

[99] O’Sullivan M., Guilford J.P. (1976). Four Factor Tests of Social Intelligence (Behavioral Cognition): Manual of Instructions and Interpretations.

[100] Myznikov A., Zheltyakova M., Korotkov A., Kireev M., Masharipov R., Jagmurov O.D., Habel U., Votinov M. (2021). Neuroanatomical Correlates of Social Intelligence Measured by the Guilford Test. Brain Topogr..

[101] Khan A.R., Hiebert N.M., Vo A., Wang B.T., Owen A.M., Seergobin K.N., MacDonald P.A. (2019). Biomarkers of Parkinson’s disease: Striatal sub-regional structural morphometry and diffusion MRI. NeuroImage Clin..

[102] Lam J.A., Murray E.R., Yu K.E., Ramsey M., Nguyen T.T., Mishra J., Martis B., Thomas M.L., Lee E.E. (2021). Neurobiology of loneliness: A systematic review. Neuropsychopharmacology.

[103] Boyes A., McLoughlin L.T., Anderson H., Schwenn P., Shan Z., Gatt J.M., Lagopoulos J., Hermens D.F. (2022). Basal ganglia correlates of wellbeing in early adolescence. Brain Res..

[104] Calderón-Garcidueñas L., Franco-Lira M., Henríquez-Roldán C., Osnaya N., González-Maciel A., Reynoso-Robles R., Villarreal-Calderon R., Herritt L., Brooks D., Keefe S. (2010). Urban air pollution: Influences on olfactory function and pathology in exposed children and young adults. Exp. Toxicol. Pathol..

[105] Calderón-Garcidueñas L., Reynoso-Robles R., Pérez-Guillé B., Mukherjee P.S., Gónzalez-Maciel A. (2017). Combustion-derived nanoparticles, the neuroenteric system, cervical vagus, hyperphosphorylated alpha synuclein and tau in young Mexico City residents. Environ. Res..

[106] Song H., Sieurin J., Wirdefeldt K., Pedersen N., Almqvist C., Larsson H., Valdimarsdóttir U.A., Fang F. (2020). Association of Stress-Related Disorders with Subsequent Neurodegenerative Diseases. JAMA Neurol..

[107] Desmarais P., Weidman D., Wassef A., Bruneau M.-A., Friedland J., Bajsarowicz P., Thibodeau M.-P., Herrmann N., Nguyen Q.D. (2020). The Interplay between Post-traumatic Stress Disorder and Dementia: A Systematic Review. Am. J. Geriatr. Psychiatry.

[108] Neylan T.C. (2020). Post-traumatic Stress Disorder and Neurodegeneration. Am. J. Geriatr. Psychiatry.

[109] Günak M.M., Billings J., Carratu E., Marchant N.L., Favarato G., Orgeta V. (2020). Post-traumatic stress disorder as a risk factor for dementia: Systematic review and meta-analysis. Br. J. Psychiatry.

[110] Jo S., Kim Y.-J., Park K.W., Hwang Y.S., Lee S.H., Kim B.J., Chung S.J. (2021). Association of NO2 and Other Air Pollution Exposures With the Risk of Parkinson Disease. JAMA Neurol..

[111] Calderón-Garcidueñas L., Stommel E.W., Rajkumar R.P., Mukherjee P.S., Ayala A. (2021). Particulate Air Pollution and Risk of Neuropsychiatric Outcomes. What We Breathe, Swallow, and Put on Our Skin Matters. Int. J. Environ. Res. Public Health.

[112] Fleury V., Himsl R., Joost S., Nicastro N., Bereau M., Guessous I., Burkhard P.R. (2021). Geospatial analysis of individual-based Parkinson’s disease data supports a link with air pollution: A case-control study. Park. Relat. Disord..

[113] Wiesman A.I., Mundorf V.M., Casagrande C.C., Wolfson S.L., Johnson C.M., May P.E., Murman D.L., Wilson T.W. (2021). Somatosensory dysfunction is masked by variable cognitive deficits across patients on the Alzheimer’s disease spectrum. eBioMedicine.

[114] Bessi V., Giacomucci G. (2021). Hidden functional derangement of somatosensory cortices in Alzheimer’s Disease. eBioMedicine.

[115] Zhang Z., Robinson L., Jia T., Quinlan E.B., Tay N., Chu C., Barker E.D., Banaschewski T., Barker G.J., Bokde A.L. (2020). Development of Disordered Eating Behaviors and Comorbid Depressive Symptoms in Adolescence: Neural and Psychopathological Predictors. Biol. Psychiatry.

[116] Jueajinda S., Stiramon O., Ekpanyaskul C. (2021). Social Intelligence Counseling Intervention to Reduce Bullying Behaviors Among Thai Lower Secondary School Students: A Mixed-method Study. J. Prev. Med. Public Health.

[117] Falgàs N., Illán-Gala I., Allen I.E., Mumford P., Essanaa Y.M., Le M.M., You M., Grinberg L.T., Rosen H.J., Neylan T.C. (2021). Specific cortical and subcortical grey matter regions are associated with insomnia severity. PLoS ONE.

[118] Ezzati A., Abdulkadir A., Jack C.R., Thompson P.M., Harvey D.J., Truelove-Hill M., Sreepada L.P., Davatzikos C., Lipton R.B., Alzheimer Disease neuroimaging Initiative (2021). Predictive value of ATN biomarker profiles in estimating disease progression in Alzheimer’s disease dementia. Alzheimer’s Dement..

[119] Jack C.R., Bennett D.A., Blennow K., Carrillo M.C., Dunn B., Haeberlein S.B., Holtzman D.M., Jagust W., Jessen F., Karlawish J. (2018). NIA-AA research framework: Toward a biological definition of Alzheimer’s disease. Alzheimer’s Dement..

[120] Jack C.R., Wiste H.J., Therneau T.M., Weigand S.D., Knopman D.S., Mielke M.M., Lowe V.J., Vemuri P., Machulda M.M., Schwarz C.G. (2019). Associations of Amyloid, Tau, and Neurodegeneration Biomarker Profiles with Rates of Memory Decline Among Individuals Without Dementia. JAMA.

[121] Tosun D., Demir Z., Veitch D.P., Weintraub D., Aisen P., Jack C.R., Jagust W.J., Petersen R.C., Saykin A.J., Shaw L.M. (2021). Contribution of Alzheimer’s biomarkers and risk factors to cognitive impairment and decline across the Alzheimer’s disease continuum. Alzheimer’s Dement..

[122] Veitch D.P., Weiner M.W., Aisen P.S., Beckett L.A., DeCarli C., Green R.C., Harvey D., Jack C.R., Jagust W., Landau S.M. (2021). Using the Alzheimer’s Disease Neuroimaging Initiative to improve early detection, diagnosis, and treatment of Alzheimer’s disease. Alzheimer’s Dement..

[123] Hampel H., Cummings J., Blennow K., Gao P., Jack C.R., Vergallo A. (2021). Developing the ATX(N) classification for use across the Alzheimer disease continuum. Nat. Rev. Neurol..

[124] Dubois B., Villain N., Frisoni G.B., Rabinovici G.D., Sabbagh M., Cappa S., Bejanin A., Bombois S., Epelbaum S., Teichmann M. (2021). Clinical diagnosis of Alzheimer’s disease: Recommendations of the International Working Group. Lancet Neurol..

