# Supplementary material for: Hemispheric Cortical, Cerebellar and Caudate Atrophy Associated to Cognitive Impairment in Metropolitan Mexico City Young Adults Exposed to Fine Particulate Matter Air Pollution
Source: Toxics. 2022 Mar 25;10(4):156. doi: 10.3390/toxics10040156 (PMC9028857; doi:10.3390/toxics10040156)
Supplement: Supplementary file 1 [file toxics-10-00156-s001.zip › toxics-1573298-supplementary.pdf]

toxics

MDPI

# Supplementary Materials: Hemispheric Cortical, Cerebellar and Caudate Atrophy Associated to Cognitive Impairment in Metropolitan Mexico City Young Adults Exposed to Fine Particulate Matter Air Pollution

**Lilian Calderón-Garcidueñas, Jacqueline Hernández-Luna, Partha S. Mukherjee, Martin Styner, Diana A. Chávez-Franco, Samuel C. Luévano-Castro, Celia Nohemí Crespo-Cortés, Elijah W. Stommel and Ricardo Torres-Jardón**

**Table S1.** Summary of MoCA cognition scores in subjects ≥31y and ≤30y in Metropolitan Mexico City MRI and non-MRI subjects and Control City Hermosillo. Adjusted p-value refers to the p-values after removing the linear effects of age, gender, BMI, and education years.

| **Residency** | **MoCA Total score Expected: 26-30** | **Trail Expected: 1** | **Cube Expected: 1** | **Clock Expected: 3** | **ID animals Expected: 3** | **Memory Expected: 5** | **Attent 1 Expected: 2** | **Attent 2 Expected: 1** | **Attent 3 Expected: 3** | **Lang 1 Expected: 2** | **Lang 2 Expected: 1** | **Abstrac Expected: 2** | **Orient 6** |
|---|---|---|---|---|---|---|---|---|---|---|---|---|---|
| All MMC ≥31 n:83 | 20.49± 3.42 | 0.79± 0.41 | 0.15± 0.36 | 1.90± 0.94 | 2.40±1.15 | 1.48± 1.45 | 1.38± 0.66 | 0.71± 0.45 | 2.10± 0.94 | 1.13± 0.77 | 0.82±0.44 | 1.58± 0.68 | 5.77± 0.45 |
| MRI MMC ≥31 n:14 | 23.36±2.84 | 0.93±0.27 | 0.43±0.51 | 2.5±0.76 | 2.79±0.80 | **2.07±1.44** | 1.64±0.63 | 0.71±0.47 | 2.36±0.93 | 1.50±0.52 | 0.93±0.27 | 1.79±0.58 | 5.79±0.43 |
| HERMOSILLO ≥31y n:13 | 25.23±2.39 | 0.92±0.28 | 0.15±0.38 | 2.00±0.91 | 2.92±0.28 | 3.31±1.75 | 2.00±0.00 | 0.92±0.28 | 2.46±0.78 | 1.92±0.28 | 0.92±0.28 | 1.69±0.48 | 5.92±0.28 |
| Adjusted p value between NON MRI MMC and controls | **< 0.0001** | 0.43 | 0.53 | 0.90 | 0.22 | **0.0004** | **0.0090** | **0.0466** | 0.49 | **0.0024** | 0.65 | 0.62 | 0.25 |
| Adjusted p value between MRI MMC and controls | **0.06** | 0.80 | 0.25 | 0.25 | 0.62 | 0.08 | 0.07 | 0.08 | 0.92 | **0.03** | 0.99 | 0.94 | 0.33 |
| MMC ≤30y n:150 | 24.27±2.62 | 0.93±0.26 | 0.57±0.50 | 2.28±0.86 | 2.97±0.27 | 2.74±1.42 | 1.69±0.48 | 0.94±0.24 | 2.43±0.84 | 1.34±0.64 | 0.86±0.37 | 1.64±0.61 | 5.81±0.44 |
| MRI MMC ≤30y n:20 | 24.50±2.63 | 0.95±0.22 | 0.65±0.49 | 2.45±0.76 | 3.00±0.00 | 2.55±1.28 | 1.65±0.49 | 0.95±0.22 | 2.35±0.93 | 1.45±0.60 | 0.85±0.37 | 1.75±0.55 | 5.75±0.44 |
| HERMOSILLO ≤30y n:22 | 24.73±2.19 | 0.95±0.21 | 0.23±0.43 | 2.14±0.71 | 3.00±0.00 | 3.14±1.39 | 1.68±0.65 | 0.95±0.21 | 2.59±0.85 | 1.27±0.55 | 1.00±0.00 | 1.86±0.35 | 5.95±0.21 |
| Adjusted p value between NON MRI MMC and controls | 0.40 | 0.82 | **0.0002** | 0.37 | 0.33 | 0.07 | 0.99 | 0.43 | 0.44 | 0.70 | **0.0414** | 0.34 | **0.17** |
| Adjusted p value between MRI MMC | 0.40 | 0.88 | **0.0009** | 0.72 | 0.62 | 0.09 | 0.70 | 0.42 | 0.20 | 0.34 | **0.08** | 0.20 | **0.03** |

| |
|---|
| and controls |

**Table S2.** Summary of MoCA scores, Cognitive Domain Scores, age, BMI and education years in MMC (MRI and NON-MRI) and Control City Hermosillo ≤30y and ≥31years. Adjusted p-value refers to the p-values after removing the linear effects of age, gender, BMI, and education years.

| Residency | MoCA scores | Average age years | BMI | Education years | Memory | EIS Total:13 Cutoff score 10.5 | LIS Total:6 Cutoff score 5.5 | VIS Total:7 Cutoff score 5.5 | AIS Total:18 Cutoff score 16 | OIS Total:6 Cutoff score 5.5 | Delay recall+EIS +VIS+LIS Total:31 Cutoff score 24 |
|---|---|---|---|---|---|---|---|---|---|---|---|
| MMC ≥31y N:83 | 20.49±3.42 | 46.4±11.8 | 27.8±3.9 | 13.2±3.3 | 1.4±1.4 | 9.2±2.2 | 4.3±1.5 | 4.4±1.71 | 15.3±1.7 | 5.7±0.4 | 19.5±4.8 |
| MRI MMC ≥31y n:14 | 23.36±2.84 | 42.7±9.3 | 28.1±4.3 | 16.0±2.1 | 2.0±1.4 | 10.8±1.4 | 5.21±1.05 | 5.71±1.38 | 17.1±1.2 | 5.7±0.4 | 23.8±3.8 |
| Hermosillo ≥31y n:13 | 25.23±2.39 | 44.0±7.2 | 26.9±4.3 | 15.2±2.8 | 3.3±1.7 | 10.9± 1.3 | 5.7±0.4 | 5.0±1.1 | 17.3±1.0 | 5.9±0.2 | 25.0±3.1 |
| Adjusted p value NON-MRI MMC vs HER | <0.0001 | NA | NA | NA | 0.0004 | 0.08 | 0.0078 | 0.44 | 0.0010 | 0.25 | 0.0005 |
| Adjusted p value MRI MMC vs HER | 0.06 | NA | NA | NA | 0.08 | 0.62 | 0.13 | 0.34 | 0.04 | 0.33 | 0.29 |
| MMC≤30y n:150 | 24.2±2.6 | 21.6±3.5 | 24.2±3.2 | 13.6±1.7 | 2.7±1.4 | 10.7±1.6 | 5.1±0.8 | 5.8±1.1 | 16.4±1.2 | 5.8±0.4 | 24.5±3.2 |
| MRI MMC ≤30y n:20 | 24.5±2.6 | 22.0±3.3 | 23.8±3.8 | 14.1±1.6 | 2.5±1.3 | 10.9±1.8 | 5.3±0.7 | 6.1±0.9 | 17.2±1.7 | 5.7±0.4 | 24.9±2.9 |
| Hermosillo ≤30y n:22 | 24.7±2.2 | 19.3±1.3 | 21.9±2.7 | 13.7±0.7 | 3.1±1.4 | 11.1±1.2 | 5.2±0.5 | 5.3±0.90 | 17.5±1.2 | 5.9±0.2 | 24.9±2.5 |
| Adjusted p value NON MRI MMC vs HER | 0.40 | NA | NA | NA | 0.07 | 0.40 | 0.43 | 0.03 | 0.65 | 0.17 | 0.48 |
| Adjusted p value MRI MMC vs HER | 0.40 | NA | NA | NA | 0.09 | 0.64 | 0.86 | 0.06 | 0.50 | 0.03 | 0.73 |

Executive Index Score (EIS) is the sum of Trail making, clock drawing, digit span forward and backward, letter A tapping, serial 7's subtraction, word fluency and abstraction.

Language Index Scores (LIS): animal naming, sentence repetition and word fluency.

Visuospatial Index Score (VIS): cube copy, clock drawing and animal naming.

Attention Index Score (AIS): digit span forward and backward, letter A tapping, serial 7s subtraction, sentence repetition and Words Recalled in Both Immediate Recall Trials.

The Orientation Index Score (OIS) includes all the Orientation items (0–6 points).

Summary Score: Delayed Recall Score plus VIS, EIS, and LIS.
